# Oxidant-assisted methane pyrolysis†

**DOI:** 10.1039/d5sc00768b

**Published:** 2025-06-03

**Authors:** Marco Gigantino, Henry Moise, Vasudev Haribal, Andrew Tong, Jian Ping Shen, Dimitri Saad, Jacob Fishman, Alexander Nelson, Harry Voorhis, Eddie Sun, Adam Brandt, Raghubir Gupta, Arun Majumdar, Matteo Cargnello

**Affiliations:** a Department of Chemical Engineering, Stanford University Stanford CA 94305 USA mcargnello@stanford.edu; b Susteon Inc. Cary NC 27513 USA; c Department of Energy Science & Engineering, Stanford University Stanford CA 94305 USA; d Department of Mechanical Engineering, Stanford University Stanford CA 94305 USA; e Precourt Institute for Energy, Stanford University Stanford CA 94305 USA; f SUNCAT Center for Interface Science and Catalysis, Stanford University Stanford CA 94305 USA

## Abstract

Methane pyrolysis has been proposed as a cost-competitive route to produce low-CO_2_-emissions hydrogen that can utilize today's infrastructure to supply feedstock and manage waste, and thereby be rapidly scalable. However, this process faces challenges such as catalyst deactivation and carbon build-up that hinder its large-scale implementation. Pyrolysis is usually conducted in the absence of oxidizers to avoid combustion products such as CO_2_. Here, we demonstrate that the addition of small concentrations of an oxidant to a methane pyrolysis reaction on Fe-based catalysts prevented catalyst deactivation and increased the net production of carbon and hydrogen. Methane pyrolysis in the presence of a small amount of CO_2_ demonstrated a twofold increase in carbon yield and a 7.5-fold increase in hydrogen concentration in the effluent compared to that of a pure methane feed during 1 h operation in a fluidized bed reactor at 750 °C. A similar beneficial effect was observed by adding small amounts of H_2_O in the feed. We provide evidence that the cyclic formation and decomposition of an iron carbide catalyst phase allowed for increased methane decomposition and significant carbon removal from the catalyst surface, thus increasing carbon and hydrogen yields. A similar result was obtained for Ni- and Co-based catalysts.

## Abstract 2

Adding small amounts of CO_2_ or H_2_O to methane pyrolysis boosts hydrogen and carbon yields *via* cyclic catalyst regeneration, enabling efficient low-carbon hydrogen and crystalline carbon production.

## Graphical abstract

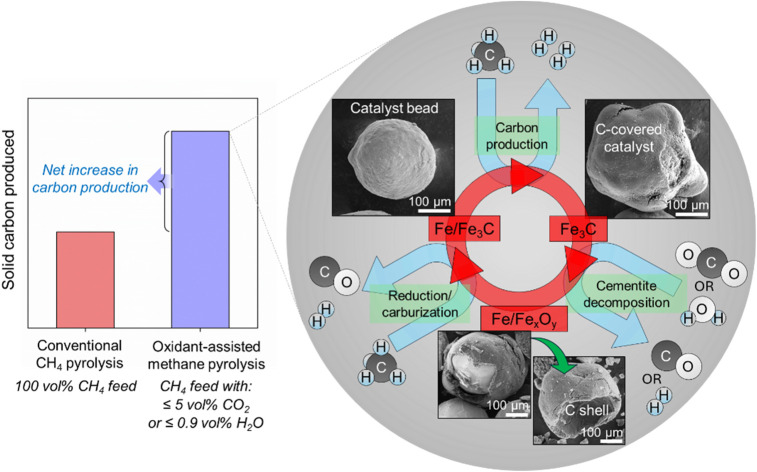

## Introduction

While there are sufficient geological reserves of coal, oil, and natural gas (NG) to fuel our society for at least the next century, the environmental consequences associated with the continued emissions of CO_2_ and other greenhouse gases (GHG) into the atmosphere have spurred a global initiative for an earlier transition away from them.^1^ The adoption of carbon taxes, emissions-based penalties and/or financial incentives for GHG-free approaches may allow for more sustainable energy sources like GHG-free hydrogen to compete in the market with fossil fuels.^2^ Additionally, advancements in technologies, such as more efficient hydrogen production methods and the co-production of valuable commodities like carbon, could further reduce costs and enhance hydrogen's competitiveness as a cleaner energy source.^3^

Currently, hydrogen is a vital chemical intermediate for many industrial sectors crucial to the global economy, including ammonia production and hydrocarbon refining.^4^ Low-cost, low-carbon hydrogen is needed to support crucial chemical processes for many decades to come. At the same time, it has the potential to scale as an advanced fuel and meet the energy demands of the primary and secondary economic sectors.^5^ Hydrogen can be utilized for power generation with zero direct emissions. Its production, however, is currently highly carbon intensive.

The most cost-effective route to produce hydrogen today is through the partial oxidation of fossil hydrocarbons *via* steam reforming or autothermal reforming followed by water-gas shift (WGS). Reforming reactors supply as much as 95% of global hydrogen demand today but do so with associated process emissions in the range of 9–12 kg CO_2_ per kg H_2_.^6^

The combination of steam methane reforming (SMR) (CH_4_ + H_2_O ⇌ 3H_2_ + CO, Δ*H*_rxn,298K_ = 69 kJ per mol H_2_) with WGS (CO + H_2_O ⇌ H_2_ + CO_2_, Δ*H*_rxn,298K_ = −41 kJ per mol H_2_) results in the stoichiometric emissions of 5.5 kg CO_2_ per kg H_2_, with the remaining emissions indirectly arising from steam generation and the high-grade heat required to sustain the SMR endothermic reaction and total up to ∼12 kg CO_2_ per kg H_2_. Oxidants play various roles in reforming processes. Steam provides half the hydrogen produced in SMR and is fed into the primary reforming reactor at mass ratios between 1.8–4 H_2_O:CH_4_.^7^ Incorporation of CO_2_ in the feed (1 CO_2_:CH_4_) allows for dry reforming of methane (DRM) (CH_4_ + CO_2_ ⇌ 2H_2_ + 2CO, Δ*H*_rxn,298K_ = 124 kJ per mol H_2_), with stoichiometric emissions of 11 kg CO_2_ per kg H_2_. Incorporation of oxygen in the feed (0.2–0.5 O_2_:CH_4_) allows for autothermal reforming (ATR) (CH_4_ + H_2_O + ½O_2_ ⇌ CO_2_ + 3H_2_, Δ*H*_rxn,298K_ = −26 kJ per mol H_2_) or partial oxidation (CH_4_ + ½O_2_ ⇌ CO + 2H_2_, Δ*H*_rxn,298K_ = −19 kJ per mol H_2_), which reduces the heat load but also increases the stoichiometric emissions (7.3 kg CO_2_ per kg H_2_ and 11 kg CO_2_ per kg H_2_, respectively).^7^

Non-oxidative conversion routes for methane have emerged as a new research opportunity for hydrocarbon utilization, including non-oxidative coupling,^8^ dehydroaromatization,^9^ and thermal decomposition or pyrolysis.^10^ Methane pyrolysis (MP) (CH_4_ ⇌ 2H_2_ + C, Δ*H*_rxn,298K_ = 37 kJ per mol H_2_) has been proposed as a cost-competitive route to produce CO_2_-free hydrogen with zero direct carbon emissions. During MP, methane decomposes in a non-oxidative environment to produce hydrogen and a solid carbon product that can either find value in the market or be sequestered in a much more stable and manageable form as compared to CO_2_. MP plants can be located where H_2_ is needed, the CH_4_ feedstock can be provided using today's natural gas pipelines and solid-carbon product can be removed using today's truck or rail infrastructure. The fact that MP is compatible with today's infrastructure and does not need scaling of new types of infrastructure makes MP rapidly scalable and cost-effective, as long as the H_2_ production costs are competitive in the H_2_ market.^11^

A key challenge for MP is the removal of the solid carbon from the reactor during the production of H_2_. The efficient removal must be carefully managed to prevent pressure build-up and catalyst loss. Several reactor configurations have been proposed to counter this challenge including fluidized bed reactors,^12^ moving bed reactors,^13,14^ molten media bubble columns,^15^ and plasma torch reactors.^16^ Sustaining high rates of methane conversion is also impeded by catalyst deactivation due to coke deposition.

This work aims to investigate whether a small, controlled amount of oxidant can prevent catalyst deactivation while maintaining the reducing environment necessary for the MP reaction to occur. To the best of our knowledge, oxidants during MP have only been used during autothermal pyrolysis (ATP) to supply *in situ* heat for the MP endothermic reaction at the expense of a decreased hydrogen and carbon yield.^17^ Feeding oxygen to supply heat *via* combustion has also been explored across multiple pyrolysis-type processes not dealing with hydrogen production such as *in situ* retorting for shale oil recovery,^18^ upgrading the heating value of low rank coals,^19^ and improving thermal degradation of biomass for biocrude and biochar production.^19,20^

Lastly, oxidants such as CO_2_,^21^ H_2_O,^22,23^ and O_2_ ^24^ have been demonstrated to enhance carbon nanotubes (CNTs) growth during chemical vapor deposition by mitigating catalyst sintering as well as selectively oxidizing amorphous carbon and annealing defects that would otherwise hinder CNTs growth. Unlike MP, chemical vapor deposition is solely optimized for CNTs growth. This focus on carbon inherently hinders its ability to also scale for hydrogen production due to expensive and dilute carbon feedstocks (typically 0.1–5 mol% of ethylene or acetylene), limited throughput to control kinetics, and low operating pressures to prevent undesired gas-phase reactions.

The addition of oxidants in MP is a seemingly counterintuitive strategy for a process theoretically designed to produce hydrogen with zero direct CO_2_ emissions, which might explain why it has been scarcely investigated. However, potential environmental concerns related to oxidant use might be alleviated. For instance, the energy consumption of a Pressure Swing Adsorption (PSA) unit for hydrogen purification is minimal compared to the energy input required in conventional hydrogen production methods, such as SMR, where high-temperature heating constitutes a major operational cost.^25^ This work demonstrates the opportunity to complement MP with controlled amounts of oxidant co-feeds, shifting operation towards oxidant-assisted methane pyrolysis (OMP).

Previously, we described a semi-continuous process to produce H_2_ and CNTs in a fluidized-bed reactor *via* repeated catalytic MP cycles that included *in situ* carbon-catalyst separation steps.^26^ In this work, we demonstrate that the addition of a dilute oxidant, namely CO_2_ or H_2_O, in the reactor feed resulted in a net increase in carbon and hydrogen yields when compared against a pure methane feed. The increased extent of methane decomposition during OMP was associated to the *in situ* cyclic phase change of the catalyst operated by the oxidant. The superior performance of OMP over conventional methane pyrolysis was demonstrated in two different reactor configurations, namely fluidized bed and monolithic reactor. In conclusion, this study introduces the route of OMP that, alongside ATP, completes the oxidative spectrum of methane utilization by bridging the gap between pyrolysis and reforming processes.

## Results and discussion

**Fig. 1:**
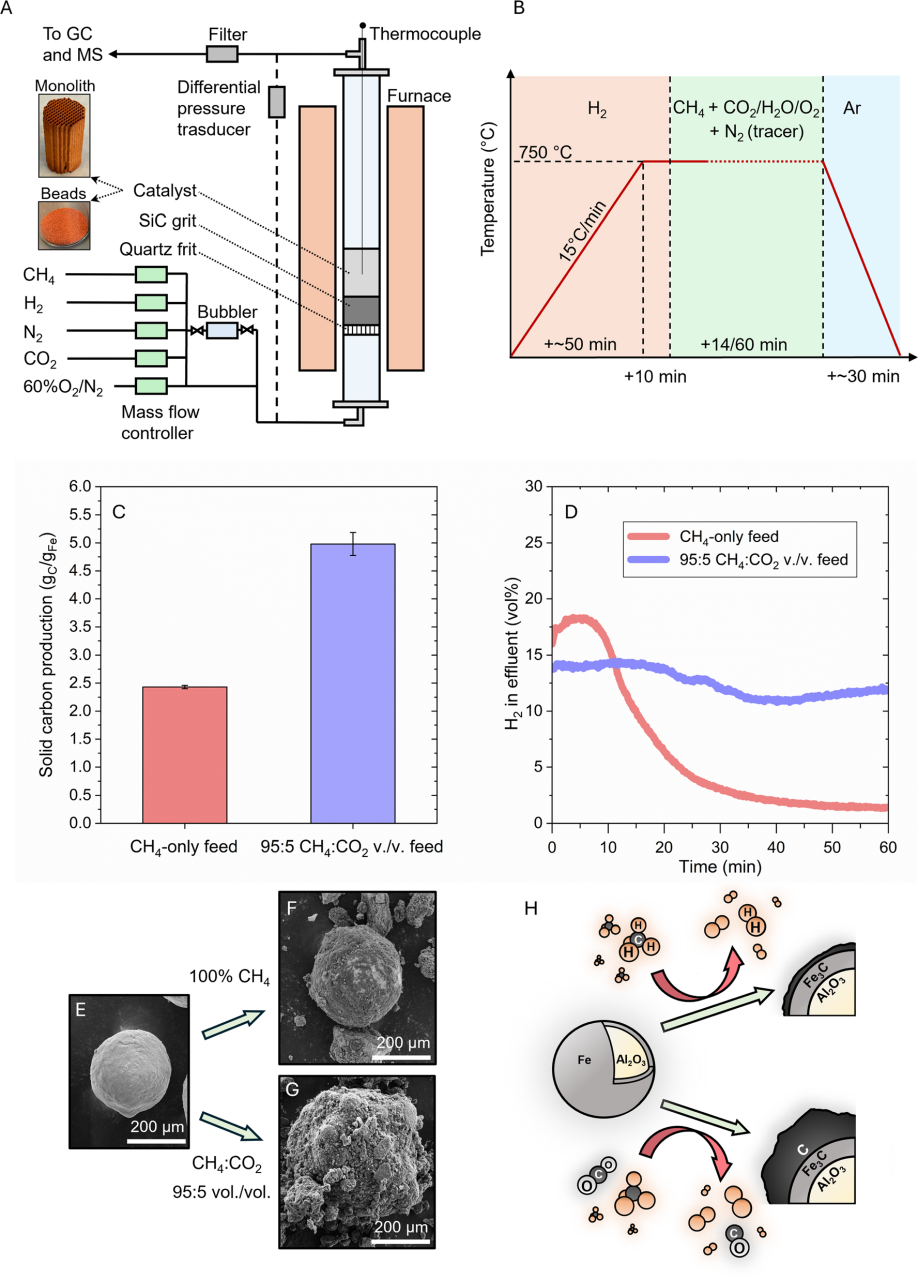
Comparison of methane pyrolysis (MP) and oxidant-assisted methane pyrolysis (OMP). (A) Schematic of the reactor set-up. (B) Graphical illustration of the reactor operation consisting of 3 steps: (i) heat-up + catalyst reduction, (ii) pyrolysis and (iii) cool-down under inert atmosphere. (C) Carbon yield, normalized by the Fe catalyst mass, and (D) hydrogen concentration in the effluent for CH_4_-only and 95 : 5 CH_4_ : CO_2_ vol./vol. reactor feed after 1 h at 750 °C (the maximum measurement uncertainty is ±1.20% of the plotted values). (E) Catalyst bead before reaction. (F) Catalyst bead after reaction under CH_4_ flow. (G) Catalyst bead after reaction under 95 : 5 CH_4_ : CO_2_ vol./vol. flow. (H) Graphical visualization of MP *versus* OMP.

Initial exploration of OMP was accomplished in a lab-scale fluidized bed reactor (FBR) operating at 750 °C with a 5 wt% Fe/Al_2_O_3_ catalyst synthesized by wet impregnation of iron nitrate on 287 μm diameter alumina beads (Fig. 1A and B). The objective was to monitor any potential increase in methane conversion along with a corresponding rise in carbon and hydrogen yield. The *in situ* reduced catalyst, exposed for 1 h to a flow of CH_4,_ displayed a total carbon yield of 2.43 ± 0.03 g_C_ g_Fe_^−1^ and a hydrogen concentration in the effluent of ∼1.4 vol% at the end of the experiment (Fig. 1C and D). The experiment was repeated by adding CO_2_ (5 vol%) as an oxidant to the gas feed mixture and resulted in a total carbon yield of 4.98 ± 0.20 g_C_ g_Fe_^−1^ and a final hydrogen concentration in the effluent of ∼11.9 vol%. The simultaneous presence of CH_4_ and CO_2_ in a 95 : 5 volume ratio resulted in a twofold increase in carbon yield and a 7.5-fold increase in hydrogen concentration in the effluent when compared to the CH_4_-only case.

A comparison of SEM images of the catalyst before and after reaction revealed thermomechanical stability of the alumina beads, which remained intact during pyrolysis, and the formation of a carbon layer on their surface (Fig. 1E–G). When CO_2_ was added to the feed, a thicker carbon layer was observed on the catalyst beads, confirming the net increase in carbon production (Fig. 1G). Some of these thick carbon shells were found to peel off from the catalyst surface, likely because of mechanical abrasion under the fluidization regime (Fig. S1†).

**Fig. 2:**
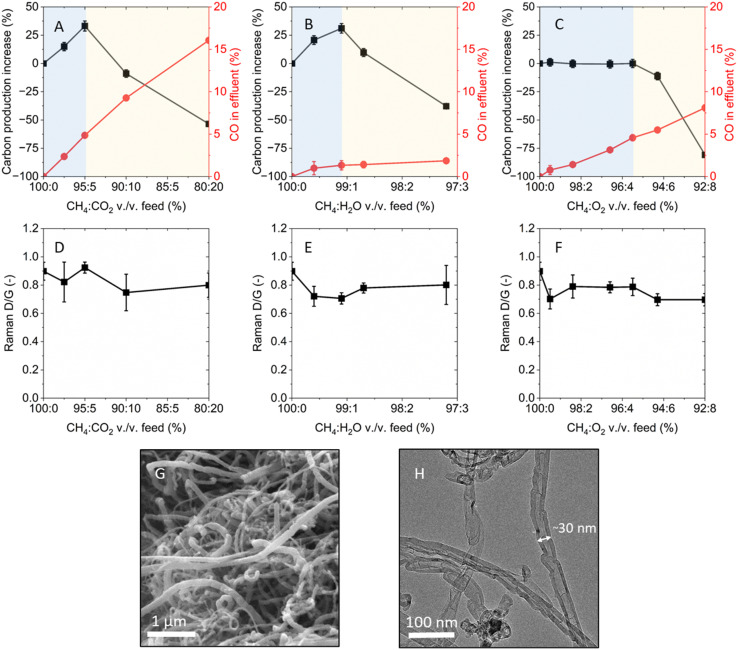
Product yield and carbon quality for different OMP conditions. Percentage of carbon produced relative to the methane-only feed experiment (left *y*-axis) and CO concentration in the reactor effluent (right *y*-axis) as a function of methane-to-oxidant vol./vol. ratio in the feed for (A) CO_2_, (B) H_2_O and (C) O_2_. On each plot, the area highlighted in blue indicates the OMP-controlled regime, while the area highlighted in yellow denotes the gasification-controlled regime. Raman *I*_D_/*I*_G_ as function of methane-to-oxidant vol./vol. ratio for (D) CO_2_, (E) H_2_O and (F) O_2_. SEM (G) and TEM (H) images of the carbon produced with a CH_4_-only feed.

We then explored whether other oxidants could lead to a similar increase in carbon yield under methane pyrolysis conditions. Selected oxidants, namely CO_2_, H_2_O, and O_2_, were individually tested in the fluidized bed reactor by incrementally increasing their concentrations in the reactants stream at 750 °C. The percentage of solid carbon produced relative to the reference case of methane-only feed and the CO concentration in the reactor effluent, both measured after 14 min of reaction, were plotted against the methane-to-oxidant volume ratio (Fig. 2A–C). The shorter duration of the experiment was used to ensure comparability in hydrodynamic conditions within the reactor as carbon was formed.

The methane-only experiment resulted in a carbon yield of 1.22 ± 0.01 g_C_ g_Fe_^−1^ (corresponding to the [0%, 0%] coordinate on the left *y*-axis of Fig. 2A–C) and no detected CO in the effluent. The addition of incremental concentrations of CO_2_ to the feed resulted in a monotonic increase in CO formation, while solid carbon production exhibited a maximum at 95 : 5 CH_4_ : CO_2_ vol./vol. (Fig. 2A). At this optimal gas feed composition, a carbon yield of 1.63 ± 0.03 g_C_ g_Fe_^−1^ was measured, which corresponded to a 34% increase from the methane-only case. Beyond the 95 : 5 CH_4_ : CO_2_ feed, the percentage of produced carbon showed a decline with increasing CO_2_ fraction, eventually resulting in negative values that indicated lower carbon formation than the methane-only case. The appearance of a peak in carbon production supports the existence of an oxidant-assisted methane pyrolysis regime prior to the onset of dominant carbon gasification. The incremental addition of CO_2_ in the methane feed was also tested with 5 wt% Ni/Al_2_O_3_ and 5 wt% Co/Al_2_O_3_ catalysts, which featured analogous behavior compared to the 5 wt% Fe/Al_2_O_3_ catalyst (Fig. S2†).

Similar trends in solid carbon and CO production were observed when using H_2_O as the oxidant. In this case, the maximum amount of carbon produced was found at a H_2_O concentration in the reactor feed corresponding to 99.1 : 0.9 CH_4_ : H_2_O vol./vol., which resulted in a carbon production of 1.60 ± 0.03 g_C_ g_Fe_^−1^ – a 31% increase compared to the methane-only case (Fig. 2B). As in the case of CO_2_, the benefits gained from H_2_O addition in low concentrations were negated at high concentrations due to lower carbon yield, likely resulting from higher rates of gasification of the carbon produced or from catalyst deactivation. CO concentration also increased with increasing H_2_O concentrations, but at a slower rate compared to the CO_2_ case.

Under increasing volume fractions of O_2_ in the reactor feed, the carbon yield did not exhibit significant change compared to the methane-only case until 95.5 : 4.5 vol./vol. CH_4_ : O_2_ (Fig. 2C). Beyond this concentration, further O_2_ addition resulted in decreasing amounts of collected solid carbon compared to the methane-only case. CO concentration in the effluent increased monotonically with O_2_ co-feed fraction.

Across all experiments performed with an oxidant in the feed, CO_2_ was not detected in the reactor effluent. Along with the CH_4_ pyrolysis reaction (*i.e.* CH_4(g)_ → C_(s)_ + H_2(g)_), solid carbon can be produced through the CO disproportionation reaction (*i.e.* 2CO_(g)_ ⇌ C_(s)_ + CO_2(g)_), also known as the Boudouard reaction, which is thermodynamically viable, though not favored under the tested conditions (Fig. S3†). The lack of measurable CO_2_ at the reactor outlet suggests that the Boudouard reaction did not contribute to the increase in solid carbon production observed.

In addition to the amount of carbon produced, the presence of an oxidant in the reactor could also affect the physical and chemical properties of the carbon. The investigated oxidants were previously shown to preferentially oxidize sp^3^-hybridized carbon (defects in graphitic carbon or amorphous carbon) over sp^2^-hybridized carbon (graphitic carbon) and, at the same time, introduce defects on graphene lattices of graphite and CNTs.^27–29^ Raman spectroscopy was used to compare the degree of crystallinity of the various carbon samples, indicated by the ratio of the D (“defective” or “disordered”, ∼1350 cm^−1^) and G (“graphitic”, ∼1580 cm^−1^) peaks intensities. The G band is associated with graphene layers, while the D band with defects and, secondarily, to amorphous carbon.

The evolution of the *I*_D_/*I*_G_ ratio at increasing oxidant-to-methane ratios in the reactor feed was measured for the individual cases of CO_2_, H_2_O, and O_2_, respectively (Fig. 2D–F). Carbon formed in presence of CO_2_ featured an average *I*_D_/*I*_G_ of 0.82 ± 0.11, very similar to the *I*_D_/*I*_G_ of 0.90 ± 0.06 of the methane-only case. Carbon grown by the addition of H_2_O in the feed featured an average *I*_D_/*I*_G_ of 0.75 ± 0.08. Similarly, the carbon formed with O_2_ in the feed featured an average *I*_G_/*I*_D_ of 0.74 ± 0.06. Overall, the *I*_D_/*I*_G_ of the various carbons fell within a narrow range, indicating comparable quality. The slightly reduced *I*_D_/*I*_G_ values obtained by the addition of an oxidant suggested that, under the tested conditions, the presence of oxidants during carbon growth could reduce the formation of amorphous carbon and, possibly, not promote the formation of defects in the graphitic carbon.

Microscopy techniques were used to examine the morphology and microstructure of the carbon produced. Scanning electron microscopy (SEM) imaging mainly showed filamentous structures (Fig. 2G), which featured similar range of length and diameter across all samples (Fig. S4†). Transmission electron microscopy (TEM) analysis revealed that the produced carbon exhibited a range of morphologies, primarily consisting of graphitic carbon, with a notable predominance of CNTs displaying a bamboo-like structure (Fig. 2H and S5†). Iron nanoparticles were found to be encapsulated inside the CNTs, as evidenced by energy dispersive spectroscopy (EDS) analysis (Fig. S6†). Common across all oxidant co-feed cases, a significant amount of carbon was found on the reactor walls, a phenomenon that was not observed in the methane-only feed (Fig. S7†). The facile separation of carbon from the catalyst surface in the form of carbon shells (Fig. S1†) demonstrated the opportunity for a more straightforward carbon removal from the reactor.

**Fig. 3:**
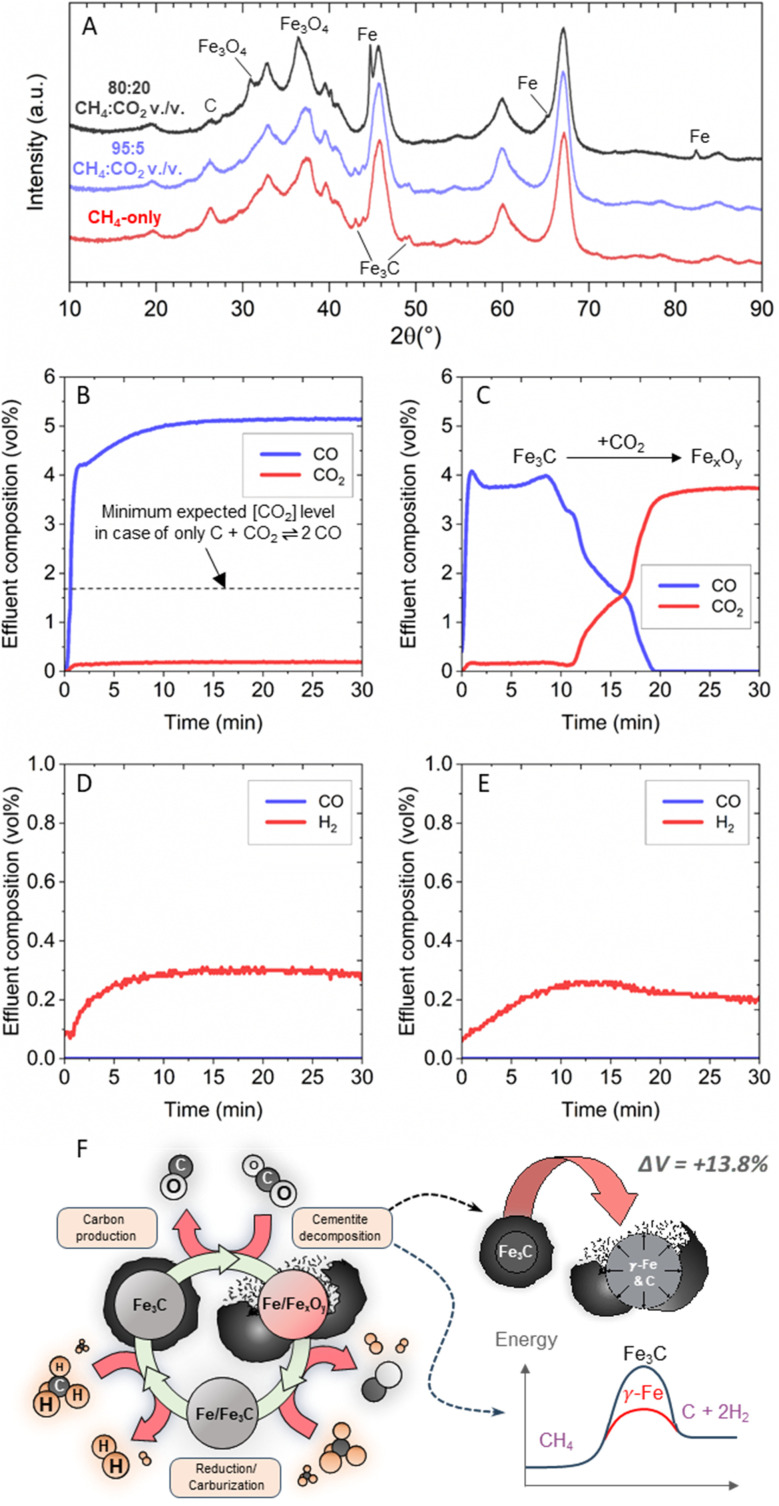
Mechanism for the enhanced catalytic activity in OMP. (A) XRD spectra of 5 wt% Fe/*θ*-Al_2_O_3_ catalysts tested under methane-only, 95 : 5 CH_4_ : CO_2_ vol./vol. and 80 : 20 CH_4_ : CO_2_ vol./vol. feed. (B) Fe_3_C and C mixture tested under a 95 : 5 Ar : CO_2_ vol./vol. feed (C) Fe_3_C tested under a 95 : 5 Ar : CO_2_ vol./vol. feed. (D) Fe_3_C and C mixture tested under a 99.1 : 0.9 Ar : H_2_O vol./vol. (E) Fe_3_C tested under a 99.1 : 0.9 Ar : H_2_O vol./vol. feed. (F) Graphical visualization of *in situ* cyclic formation-decomposition of cementite.

The increase in solid carbon yield observed with the addition of CO_2_ and H_2_O in low concentrations was lost at higher concentrations (Fig. 2A and B). *Ex situ* X-ray Diffraction (XRD) analysis of the spent catalysts of the CO_2_ co-feed case provided insights into the potential mechanism behind the observed change in activity at increasing oxidant concentration (Fig. 3A). A consistent trend was observed across the H_2_O and O_2_ co-feeds cases as well (Fig. S8 and S9†). Cementite (Fe_3_C) peaks at 43.7°, 45.1°, 48.6°, and 49.0° were the only distinguishable iron phase peaks present for the methane-only feed, as well as every oxidant co-feed which resulted in a net positive increase in solid carbon production. At oxidant concentrations associated with a decrease in solid carbon production, namely 80 : 20 CH_4_ : CO_2_ vol./vol. (Fig. 3A), the cementite phase peaks were lost and replaced by a combination of metallic iron (peaks 44.7°, 65.0°, and 82.3°) and magnetite (Fe_3_O_4_, peaks 30.5° and 36.5°). Cementite phase loss was also observed for both the 97.2 : 2.8 CH_4_ : H_2_O vol./vol. and 92 : 8 CH_4_ : O_2_ vol./vol. feed samples, which resulted in mixtures of magnetite, wüstite (FeO), and reduced metallic iron (Fig. S8 and S9†).

Iron shows two stable crystal structures depending on temperature: the face-centered cubic (FCC) austenite (γ-iron) phase and the body-centered cubic (BCC) ferrite (α-iron) phase. The conditions used in this work were above the threshold temperature for α-to-γ Fe phase transition (723 °C for >0.02 wt% C), and austenite was expected to be the thermodynamically stable state of iron. Its FCC structure allows for higher carbon solubility and the formation of carbide species.^30^ Cementite is frequently reported as the active catalyst phase during methane decomposition,^31–33^ though some studies suggest metallic iron is more active than cementite near our operating temperature.^34^ Regardless of cementite activity, it is an ensuing intermediate phase of methane decomposition as iron is a carbide-forming metal and any fully dehydrogenated carbon atoms chemisorbed on its surface will diffuse into the catalyst bulk.^35^ The presence of oxidized catalyst phases at higher oxidant co-feed concentrations may explain the observed loss of activity. This also suggests that catalyst oxidation may be occurring to some degree at the lower oxidant concentrations leading to an increase in solid carbon production, potentially shedding light on the mechanism of OMP.

To investigate in more detail the interactions between the oxidant and the surface of the catalyst that led to increased carbon production, the methane and oxidant feeds were separated into two consecutive stages to probe their individual effects on the catalyst phase and composition. Pyrolysis with pure methane was performed for 10 min at 750 °C to produce cementite phase and carbon. The reactor was then purged with argon to remove hydrogen and unreacted methane. The optimized 95 : 5 CH_4_ : CO_2_ vol./vol. feed was then introduced into the reactor at the same flow rates employed during OMP operation but using argon instead of methane while tracking effluent concentration (Fig. 3B). The only relevant species leaving the reactor were CO and CO_2_ at concentrations that indicated a CO_2_ conversion of approximately 93%. This conversion exceeded the thermodynamic limit of 64% expected if CO_2_ reacted solely with the carbon product *via* the reverse Boudouard reaction (Fig. S3†), indicating that the CO_2_ must also have reacted with the catalyst.

To further investigate the role of catalyst reactivity with CO_2_, a pure cementite catalyst phase without carbon was successfully produced by treating the catalyst under a pure methane feed at 500 °C for 1 h prior to reaction following a previous published work (Fig. S10†).^36^*Ex situ* XRD demonstrated that this produced cementite phase was stable even after 30 min at 750 °C under argon, as well as after exposure to ambient air during transport to the XRD instrument. The experiment outlined previously using 95 : 5 Ar : CO_2_ vol./vol. was then replicated using this pure cementite catalyst phase. Sustained CO_2_ conversion into CO was observed for ∼10 min before declining until a complete loss of CO detection occurred at ∼20 min (Fig. 3C). *Ex situ* XRD revealed that the cementite phase was completely converted into magnetite and wüstite phases after reaction with CO_2_ (Fig. S11†).

The initial effluent concentration was comparable between the experiments starting with either cementite and carbon (Fig. 3B) or only cementite (Fig. 3C). This result suggested that CO_2_ mostly reacted with the cementite phase as opposed to reacting with carbon in the reverse Boudouard reaction and the sustained activity in Fig. 3B was due to the presence of excess carbon capable of regenerating the cementite phase. The presence of iron oxide species on the spent catalyst from Fig. 3C indicated that CO_2_ oxidized metallic iron without the presence of either carbon or methane to regenerate the lost cementite phase (Fig. S11†).

The experiments above were reproduced using the optimized 99.1 : 0.9 CH_4_ : H_2_O vol./vol. feed to also understand the interaction between the H_2_O co-feed and the catalyst surface. The reactor effluent from a catalyst initially containing both cementite and free carbon was tracked (Fig. 3D), as well as the reactor effluent using a pure cementite phase catalyst (Fig. 3E). Interestingly, the only product observed in both experiments was hydrogen, with no detection of CO. Unlike the CO_2_ co-feed, which can only produce CO in all its reactions with cementite, carbon, and metallic iron, the product distribution observed under a H_2_O co-feed is highly dependent on which reaction it participates in. The lack of measurable CO_*x*_ in the reactor effluent indicated that H_2_O could not have reacted with any carbonaceous species, but instead only reacted with the iron in the cementite phase. This observation also suggested that the CO evolution observed during OMP with the 99.1 : 0.9 CH_4_ : H_2_O vol./vol. feed (Fig. 2B) must have resulted from methane acting as the reductant for the iron oxide produced *in situ* (Fig. S12†).

Given that in both the 95 : 5 CH_4_ : CO_2_ vol./vol. and 99.1 : 0.9 CH_4_ : H_2_O vol./vol. feed the oxidants readily reacted with cementite, and that cementite was the only iron phase detected on the spent catalysts (Fig. 3A and S8†), we conclude that cyclic formation-decomposition of cementite was occurring *in situ* and explained the increase in carbon production during OMP. As a metastable species at 750 °C, there exists a thermodynamic driving force for cementite to either undergo a reaction or decompose into a more stable state of austenite and graphite. The limited stability of cementite is contingent on the highly reducing methane atmosphere and the source of carbon imparted by methane decomposition. While carbon gasification and dry reforming rates over Fe catalysts are sufficiently low at 750 °C,^37,38^ small concentrations of mild oxidants like CO_2_ and H_2_O may be able to selectivity oxidize cementite due to its inherent instability at this temperature.

The oxidation of either iron or carbon in the cementite lattice shifts the phase equilibria, which may accelerate cementite decomposition as its stability is highly dependent on the local concentrations of iron and carbon.^39^ The decomposition of cementite into austenite and graphite results in a material volume expansion of 13.8%,^39^ which may be capable of delaminating the carbon shells encapsulating the catalyst. This hypothesis may explain the significant amounts of dislodged carbon found in the catalyst bed and lining the reactor walls after the reaction.

In summary, the enhanced methane conversion observed under the CO_2_ and H_2_O co-feeds may be explained by two different processes (Fig. 3F). On one side, the dislodgement of carbon from the catalyst surface occurs because of phase change that regenerated the active sites. On the other side, metallic iron formed from the decomposition of cementite may be a more active catalyst for methane decomposition and the mild oxidants helped suppress cementite formation.^34^ These processes may be occurring synergistically.

**Fig. 4:**
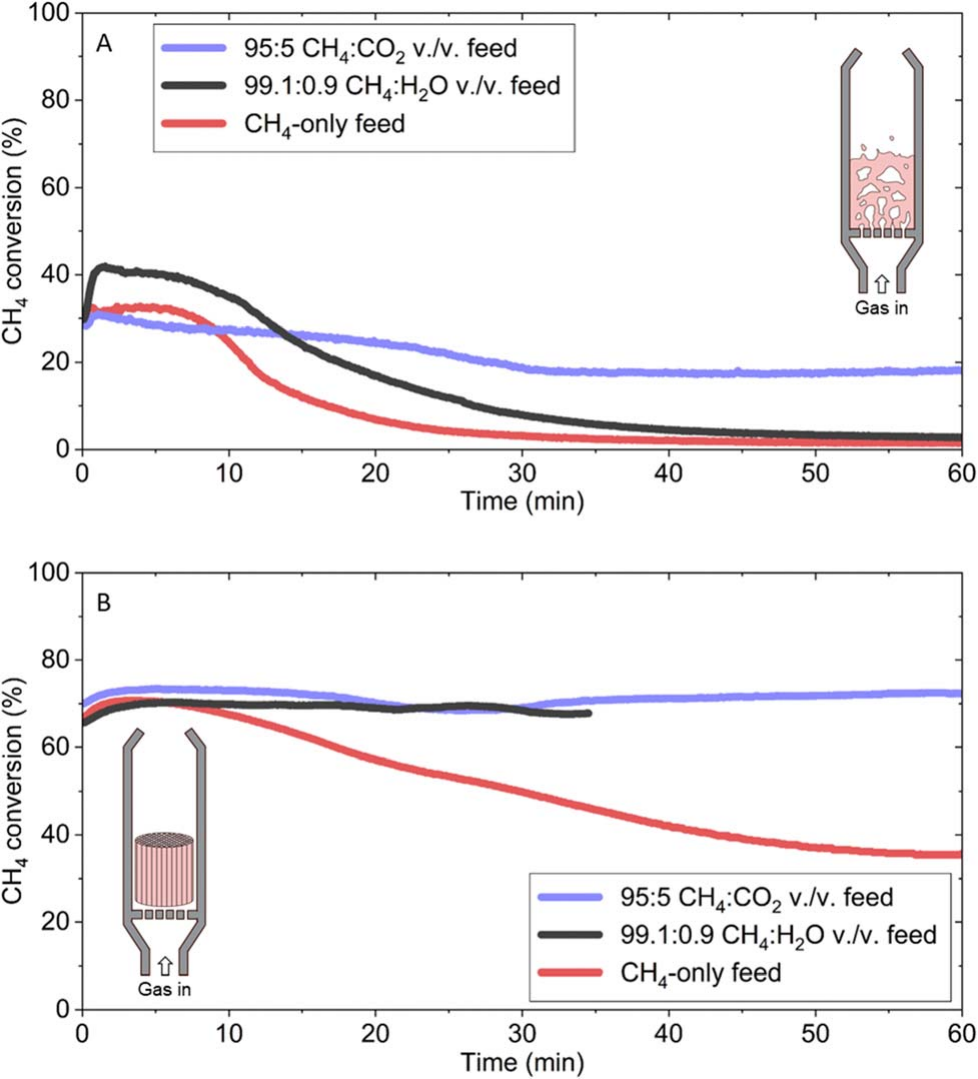
Comparison of OMP *versus* MP performance in different reactor configurations. Methane conversion as function of time in (A) fluidized-bed and (B) monolithic reactor. The maximum measurement uncertainty is ±1.20% of the plotted values for both the CH_4_-only and 95 : 5 CH_4_ : CO_2_ vol./vol. feeds in both reactor configurations, while for the 99.1 : 0.9 CH_4_ : H_2_O vol./vol. feed it is ±6.36% of the plotted values in the FBR, and ±1.40% of the plotted values in the monolithic reactor.

To gauge the overall performance of CO_2_ and H_2_O co-feeds at their optimal concentrations, methane conversion in the FBR was tracked for each condition with a weight hourly space velocity (WHSV) of ∼14.5 h^−1^ and compared against a methane-only feed (Fig. 4A). Oxygen was not included because it introduced emissions without increasing solid carbon production. All three experiments started with a methane conversion of ∼30% followed by a decline and stabilization at a pseudo-steady state conversion. Methane conversion for the 95 : 5 CH_4_ : CO_2_ vol./vol. feed performed the best with a steady-state conversion at 1 h of 18.1%, followed by 2.8% for the 99.1 : 0.9 CH_4_ : H_2_O vol./vol. feed and 1.5% for the methane-only feed. This trend in methane conversion also correlated with solid carbon production of 4.98 ± 0.20 g_C_ g_Fe_^−1^, 2.89 ± 0.25 g_C_ g_Fe_^−1^, and 2.43 ± 0.03 g_C_ g_Fe_^−1^, respectively.

As was commonly observed across all OMP experiments, both the 95 : 5 CH_4_ : CO_2_ and 99.1 : 0.9 CH_4_ : H_2_O vol./vol. feeds featured substantial accumulations of carbon within the bed and along the reactor walls, whose dislodgement from the catalyst surface was likely aided by fluidization-induced abrasion. Fluidized catalytic cracking units also suffer from attrition,^40^ making it reasonable to assume that it provides a means for carbon separation and removal with a cost-effective catalyst. As gas–solid separation and removal was not the focus of this work, the produced carbon was allowed to accumulate in the bed during operation.

The discrepancy in methane conversion observed between the 95 : 5 CH_4_ : CO_2_ and 99.1 : 0.9 CH_4_ : H_2_O vol./vol. feed may be explained by the different hydrodynamics each system experienced during operation. It is well known that the hydrodynamics and performance of a fluidized bed reactor are highly dependent on many parameters that are inherently dynamic in methane pyrolysis. Specifically, the solid carbon production that results from methane decomposition changes the diameter, density, and sphericity of the fluidized particle, all of which directly affect fluidization with a compounding effect on performance.^41^

Additionally, the CO_2_ co-feed conditions resulted in the emission of almost four times more CO than the H_2_O co-feed (Fig. 2A and B). This increase in CO production must ultimately result from the consumption of methane feedstock, which means that the CO_2_ co-feed did not produce as much net solid carbon as an H_2_O co-feed for the same methane conversion. The higher initial rate of carbon deposition under the 99.1 : 0.9 CH_4_ : H_2_O vol./vol. feed meant that the two systems experienced different rates of changes to their hydrodynamics. This observation may partially explain why methane conversion under the 99.1 : 0.9 CH_4_ : H_2_O vol./vol. feed was lower than that of the 95 : 5 CH_4_ : CO_2_ vol./vol. feed (Fig. 4A).

To avoid the influence of hydrodynamics on methane decomposition as well as assess how effectively OMP could be translated to other reactor configurations, the process was tested in a monolithic reactor configuration with the same WHSV of ∼14.5 h^−1^ as the FBR. The monolithic reactor helped reduce pressure drop across the bed and increased heat and mass transfer rates. This design was particularly advantageous for methane decomposition processes compared to traditional packed bed reactors as its larger open area allowed for more carbon accumulation before clogging and pressure build-up events.

The main benefit of using the monolithic reactor in this study was to fairly compare the two oxidant co-feeds under similar hydrodynamic environments. Using Fe/Al_2_O_3_ catalyst wash-coated on the monolithic cordierite substrate, an initial methane conversion greater than 65% was observed for both the 95 : 5 CH_4_ : CO_2_ and 99.1 : 0.9 CH_4_ : H_2_O vol./vol. feeds (Fig. 4B). The quality of the carbon formed in the monolithic reactor was comparable to that from the FBR (Fig. S13†). The 99.1 : 0.9 CH_4_ : H_2_O vol./vol. feed accumulated carbon at a higher rate than the other two conditions, which led to clogging and an ensuing pressure increase which required the experiment to be stopped at an earlier time. The pure methane feed also started at the same initial methane conversion, but slowly decreased to ∼35% by the end of the experiment demonstrating that OMP outperformed MP in both the FBR and monolithic reactor.

The experimental data from the monolithic reactor were utilized to assess the potential economic advantages of integrating OMP processes into a gas turbine power plant. A preliminary technoeconomic analysis (TEA) of this case study suggested that the OMP-integrated process could result in lower electricity costs compared to the integration of conventional MP or direct methane combustion, mainly due to the additional revenue from selling the co-produced solid carbon (details of the TEA are presented in the ESI†). This application of OMP exemplifies the potential of this approach in advancing the clean energy transition. Pipeline-quality natural gas typically contains up to ∼4 vol% CO_2_ and water vapor concentrations up to ∼0.17 vol%.^42,43^ These values correspond with the lower end of the beneficial oxidant concentrations reported in this study. This alignment suggests that the oxidant-assisted methane pyrolysis approach presented here could be implemented directly with natural gas streams, without the need of pre-treatment. Furthermore, the process may offer a route to valorize natural gas resources with higher oxidant content that are currently underutilized due to processing costs and emissions constraints.

## Conclusions

In this study, the concept of oxidant-assisted methane pyrolysis (OMP) was introduced. At first, it was demonstrated that the addition of a small amount of CO_2_ to a methane feed entering a fluidized bed of Fe/Al_2_O_3_ catalyst at 750 °C resulted in a two-fold increase in carbon yield compared to methane-only feed over 1 h operation, with a corresponding increase in hydrogen yield and producing CO as a side product. The presence of CO_2_ enabled the formation of a thick carbon layer on the catalyst beads prone to detachment under fluidization-induced abrasion. The introduction of other oxidants, such as H_2_O and O_2_, was also investigated. Optimal oxidant-to-methane ratios for carbon formation were identified for both CO_2_ and H_2_O, beyond which gasification became dominant. Peak carbon yields were measured for 95 : 5 CH_4_ : CO_2_ vol./vol. and 99.1 : 0.9 CH_4_ : H_2_O vol./vol. feeds, which resulted in a carbon production increase of 34% and 31%, respectively, compared to the methane-only feed over 14 min operation. Similar results were also observed for Ni/Al_2_O_3_ and Co/Al_2_O_3_ catalysts tested with a CO_2_ co-feed under the same conditions. Raman spectroscopy indicated that oxidant addition slightly promoted the removal of amorphous carbon, enhancing the accumulation of graphitic carbon in the product. Microscopy techniques revealed the predominant formation of carbon nanotubes (CNTs) across all conditions.

Cementite (Fe_3_C) was identified as a crucial intermediate for methane decomposition, with experiments evidencing its cyclic formation and decomposition when CO_2_ and H_2_O were introduced. The decomposition of cementite, accelerated by the oxidant, enhances the production of carbon by facilitating the regeneration of active sites on the catalyst surface. These findings reveal a dynamic process in which catalyst phases are continually evolving. At high oxidant concentrations, the transition from cementite to iron oxides accounts for the observed decline in catalyst activity and highlights the importance of optimizing oxidant levels for the effective operation of methane pyrolysis.

Comparative studies in both fluidized bed and monolithic reactor configurations confirmed that methane conversion rates and solid carbon accumulation were higher under CO_2_ and H_2_O co-feeds compared to the methane-only feed, validating the effectiveness of OMP across different reactor types.

Overall, this study highlights the potential of oxidant-assisted methane pyrolysis as an innovative approach to enhance catalyst performance and product yields relative to conventional methane pyrolysis, paving the way for more efficient production of hydrogen and crystalline carbon.

## Methods

### Catalysts preparation

The catalyst beads used in the fluidized bed reactor configuration were prepared using 287 μm avg. diameter beads made of *θ*-Al_2_O_3_ (Puralox 300/130 from Sasol), sieved to remove the fraction below 250 μm. Fe(NO_3_)_3_·9H_2_O (99+%, for analysis from Thermo Fisher Scientific) was dissolved in deionized water and the resulting solution was added dropwise to the Al_2_O_3_ beads, shaken by a vortex mixer, to obtain a 5 wt% Fe loading on the total final catalyst mass. The slurry was dried for approx. 2 h in a rotary evaporator (60 °C at 20 rpm and 20 mbar) then calcined in air at 450 °C for 5 h, with heating and cooling at 2 °C min^−1^. After calcination, the catalyst was sieved to remove residual loose powder and stored in a vacuum desiccator until usage. The catalyst was reduced *in situ* by heating up to 750 °C at 15 °C min^−1^ under a 500 sccm H_2_ flow and holding at those conditions for 10 min. Some experiments required Fe_3_C as the initial catalyst phase, which was prepared *in situ* following the procedure described by Pilipenko *et al.*^36^ As-synthesized catalyst was reduced under H_2_ at 750 °C then cooled down to 500 °C, where it was isothermally carburized for 1 h under a 285 sccm CH_4_ flow. Afterwards, the system was brought back to the reaction temperature of 750 °C under Ar. XRD analysis confirmed the presence of Fe_3_C and the absence of free carbon both at the end of *in situ* carburization and after 30 min at 750 °C under Ar, which proved that Fe_3_C does not thermally decompose (Fig. S10†).

Monolithic catalysts were prepared from a cordierite honeycomb substrate (400 cspi from Corning). The original 6 × 6′′ monolith substrate was cut with a hole saw to obtain 0.75′′-dia. cylindrical pieces of ∼1.5′′ length. The substrate pieces were dried for 2 h in a vacuum drying oven (80 °C at −25 in Hg) and then coated with a porous layer of Al_2_O_3_ (Fig. S14†). For the coating, a slurry was prepared by mixing 20 wt% of Al_2_O_3_ powder (Puralox SCFa140 UF3 from Sasol, with a 138 m^2^ per g SSA) jet-milled down to an average particle diameter of 3.5 μm, with 5 wt% of uncalcined bohemite binder (Disperal P2 from Sasol) and 75 wt% of deionized water. The slurry was vigorously shaken for 10 min, then its pH adjusted to 3.5 with the addition of acetic acid (glacial, from Fisher Chemical), and finally shaken again for 10 min. The monolith substrates were submerged into the slurry for 1 min, then passed under a sheet of high-velocity dry air created with an air knife (air pressure set at 70 psi). The passes were alternated on both sides until all excess slurry was blown away and a thin layer was left. The slurry-coated monoliths were dried for 2 h in a vacuum drying oven (80 °C at −25 in Hg), then calcined in air at 870 °C for 4 h with 2 °C min^−1^ as heating and cooling ramp. The Al_2_O_3_ coating procedure (submersion in slurry, removal of excess slurry, drying and calcination) was performed once again to deposit another layer of porous Al_2_O_3_ before the Fe catalyst loading. Al_2_O_3_-coated monoliths were submerged into a 1 M solution of Fe(NO_3_)_3_·9H_2_O in deionized water for 30 min, then passed under a sheet of high-velocity dry air (air pressure set at 40 psi), alternating both sides, to remove excess solution. The nitrate-solution-loaded monoliths were dried for 2 h in a vacuum drying oven (80 °C at −25 in Hg), then calcined in air at 450 °C for 5 h with 2 °C min^−1^ as heating and cooling ramp. The Fe loading procedure (submersion in nitrate solution, removal of excess solution, drying and calcination) was repeated 5 times to load ∼7–8 wt% Fe on the total catalyst mass.

### Reactor setup and performance

Pyrolysis experiments were carried out in a bench-scale reactor set-up, customizable for either fluidized or monolithic bed configuration. A fritted, 20-mm-ID, 25-mm-OD, quartz tube (from Prism Research Glass) was vertically positioned in an electrical furnace with a 440-mm-long heating zone (from MTI Corp). A 5-cm-long bed of SiC grit (from Kramer Industries) was used to pre-heat and distribute the gas entering the reactive zone. In the fluidized bed configuration, the reactive bed consisted of 5 wt% Fe/*θ*-Al_2_O_3_ catalyst. 14.3 g of as-produced catalyst beads were used for the 14-min-long experiments, while 17.0 g for the 1-h-long ones. In the monolith bed configuration, two 0.75′′-diameter, 1.5′′-long Al_2_O_3_-coated monoliths with a ∼7–8 wt% Fe loading and total weight of ∼5 g were placed on top of each other. A K-type thermocouple (from Omega Engineering, Inc.) was placed right at the top of the reactive zone to monitor its temperature, while a differential pressure transducer (from Omega Engineering, Inc.) was used to measure the pressure across the reactor. Mass flow controllers (model DPC17 from Aalborg) were used to flow the gases (99.999%, from Airgas). The desired amount of H_2_O was introduced in the reactor by flowing the appropriate amount of dry gas through a glass bubbler filled with deionized water at ambient temperature. The water saturation level of the gas was measured with off-line measurements using a humidity sensor (HMT120 from Vaisala) and was typically ∼80%. The gas exiting the reactor was filtered with a 2-μm-pore-size paper filter (from Savillex Corp.) before being sampled by a mass spectrometer (HPR-20 R&D from Hiden Analytical), and a gas chromatographer (from SRI Instruments) equipped with both a thermal conductivity detector and flame ionization detector with a methanizer. In a typical run, the reactor was heated up to 750 °C at 15 °C min^−1^ under a 500 sccm H_2_ flow and held at those conditions for 10 min to reduce the catalyst. Next, in the case of fluidized bed configuration, a 285 sccm CH_4_ flow was supplied together with the appropriate flow of oxidizer (CO_2_, H_2_O or O_2_) to give the desired feed composition for pyrolysis. The flowrates during both reduction and pyrolysis were chosen to be approximately twice the minimum fluidization velocity measured for each gas. A N_2_ flow was also fed to the reactor to serve as an inert tracer for gas composition analysis. 50 sccm N_2_ were used for the 14-min-long experiments and 110 sccm N_2_ for the 1-h-long ones. In the case of monolithic bed configuration, the only difference consisted in the value of CH_4_ and oxidizer flow rates, which were adjusted to match the same weight hourly space velocity (WHSV, calculated as mass flow rate of CH_4_ divided the mass of Fe) of the 1-h-long experiments in the fluidized-bed reactor configuration. A CH_4_ flow between 120 and 130 sccm (adjusted within this range depending on the monolith weight) was supplied together with the appropriate flow of oxidizer (CO_2_, H_2_O or O_2_) to give the desired feed composition. After the reaction stage, a 200 sccm Ar flow was supplied to prevent further reactions while cooling down to room temperature. After the experiment, the mixture of catalyst and produced carbon was separated from the SiC grit by sieving. The CH_4_ conversion was calculated as
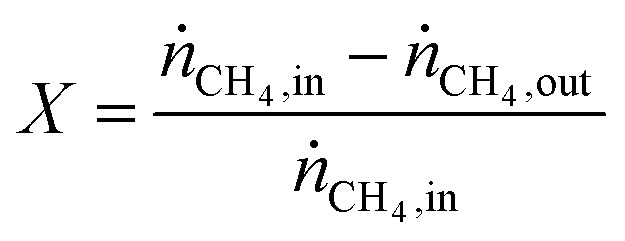
where *ṅ*_CH_4_,in_ and *ṅ*_CH_4_,out_ are the inlet and outlet CH_4_ molar flow rates, respectively. While the first is a known value, the second was calculated by comparing the measured outlet concentration against a calibration curve, which was derived with concentrations measured while flowing the same N_2_ flow rate as in the experiments.

### Materials characterization

The catalyst and the produced carbon were characterized with a series of analytical techniques. X-ray diffraction (XRD) analysis was performed with a Rigaku MiniFlex 600 X-ray diffractometer using a copper K-α radiation source (*λ* = 1.5405 Å) operated at 40 kV and 15 mA. Scanning electron microscopy (SEM) images were obtained using a Thermo Fisher Scientific Apreo S LoVac microscope, with detection of both secondary and backscattered electrons. Raman spectroscopy was performed using a HORIBA Scientific XploRA+ Confocal Raman spectrometer with a 532-nm laser source. Transmission Electron Microscopy (TEM) images were collected with a Thermo Fisher Scientific Spectra 300 transmission electron microscope with a field-emission gun operating with an accelerating voltage of 300 kV. A SuperX energy dispersive spectroscopy (EDS) detector integrated in the transmission electron microscope was used to perform elemental analysis. Thermodynamic data were derived using the FactSage 8.3 database.

## Author contributions

M. G., H. M., V. H., A. T., J. P. S., E. S., R. G., A. M. and M. C. designed the project. M. G., H. M., J. F., A. N. and H. V. synthesized the catalyst. M. G. and H. M. performed the pyrolysis experiments and characterizations. M. G., D. S. and A. B. conducted the technoeconomic analysis. A. M. and M. C. supervised the project. M. G. and H. M. wrote the manuscript, with input and revisions by all authors.

## Conflicts of interest

A provisional patent with the findings reported in this work was filed by Susteon Inc. and Stanford University.

## Supplementary Material

SC-016-D5SC00768B-s001

## Data Availability

The authors declare that the data supporting the findings of this study are available within the article and the ESI.†
