# Supplementary material for: Oxidant-assisted methane pyrolysis
Source: Chem Sci. 2025 Jun 3;16(29):13256–66. doi: 10.1039/d5sc00768b (PMC12183648; doi:10.1039/d5sc00768b)
Supplement: SC-016-D5SC00768B-s001 [file SC-016-D5SC00768B-s001.pdf]

Supplementary Information (SI) for Chemical Science.
This journal is © The Royal Society of Chemistry 2025

# Supporting Information

## Oxidant-assisted methane pyrolysis

Marco Gigantino,[1,#] Henry Moise,[1,#] Vasudev Haribal,[2] Andrew Tong,[2] Jian Ping Shen,[2] Dimitri Saad,[3] Jacob Fishman[1], Alexander Nelson[1], Harry Voorhis[1], Eddie Sun[4], Adam Brandt,[3] Raghubir Gupta,[2] Arun Majumdar[4,5] and Matteo Cargnello[1,6,*]

[1] *Department of Chemical Engineering, Stanford University, Stanford, CA 94305, USA.*

[2] *Susteon Inc., Cary, NC 27513, USA*

[3] *Department of Energy Science & Engineering, Stanford University, Stanford, CA 94305, USA.*

[4] *Department of Mechanical Engineering, Stanford University, Stanford, CA 94305, USA.*

[5] *Precourt Institute for Energy, Stanford University, Stanford, CA 94305, USA*

[6] *SUNCAT Center for Interface Science and Catalysis, Stanford University, Stanford, CA 94305, USA.*

[#] *These authors contributed equally.*

*† Electronic supplementary information (ESI) available.*

* *Corresponding author e-mail: mcargnello@stanford.edu*

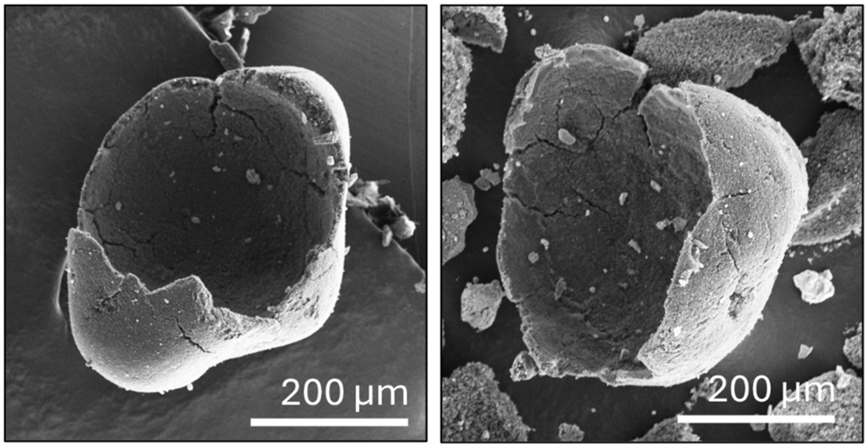


**Figure S1.** SEM images of carbon shells peeled off from catalyst beads, produced under a 95:5 $CH_4$:$CO_2$ vol./vol. feed.

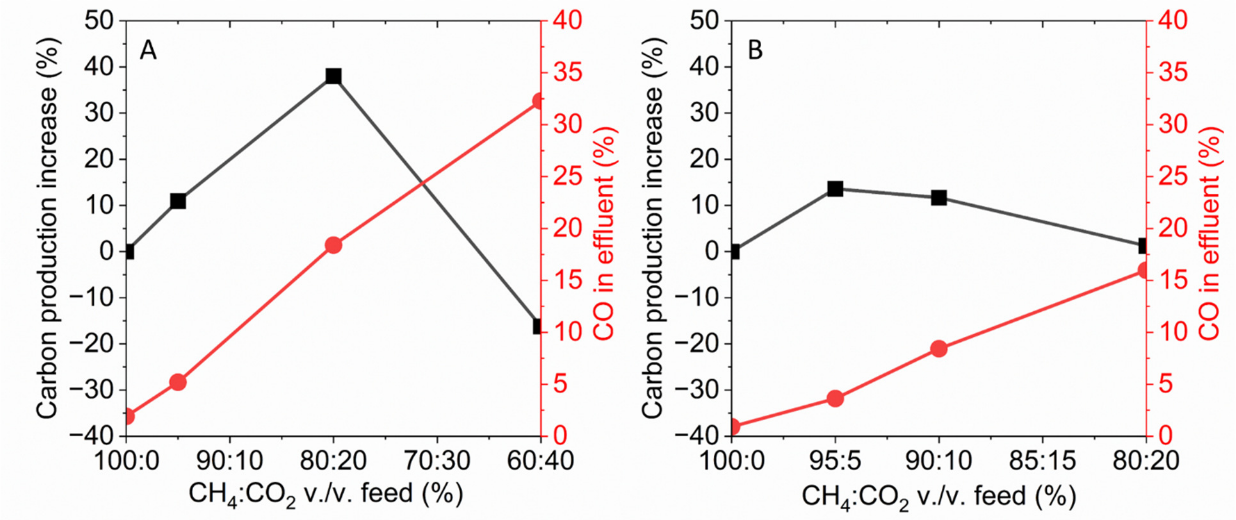


**Figure S2.** Percentage of carbon produced relative to the base-case experiment with $CH_4$-only feed and CO concentration in the reactor effluent as function of $CH_4$-to-$CO_2$ vol./vol. in the feed on (**A**) 5 wt. % $Ni/Al_2O_3$ and (**B**) 5 wt. % $Co/Al_2O_3$ catalysts.

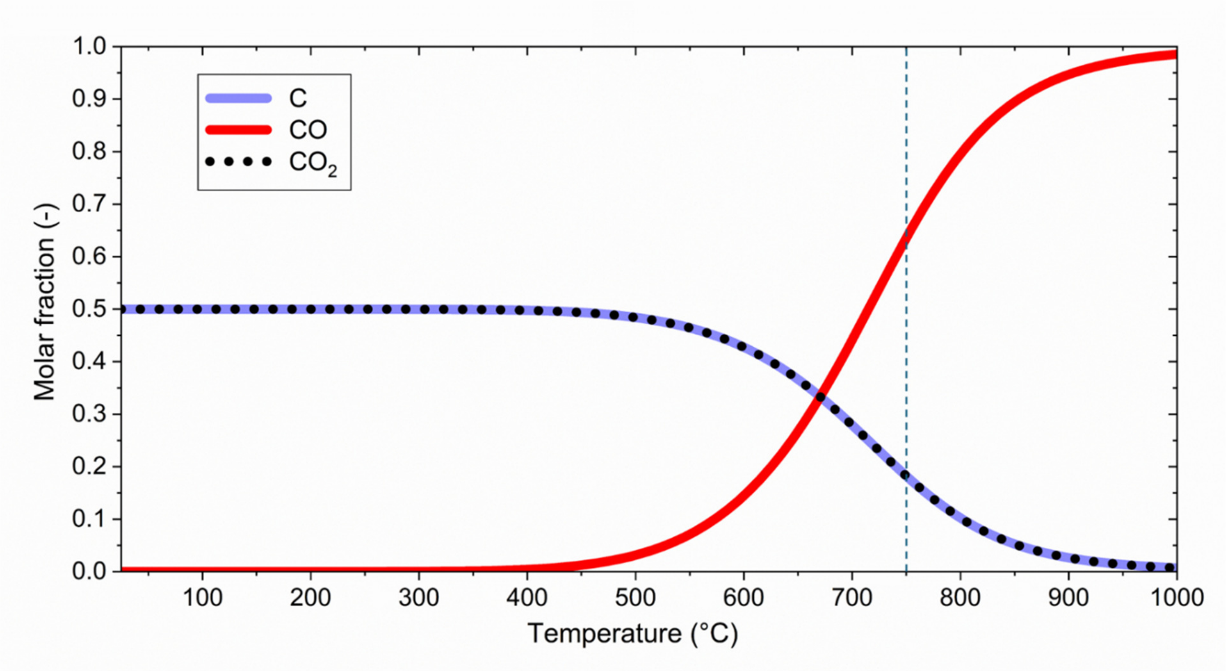


**Figure S3.** Equilibrium molar composition as function of temperature for an initial mixture of 1 mol of C (graphitic) and 1 mol of $CO_2$ at constant pressure of 1 atm. The vertical dashed line indicates the operating temperature of this work.

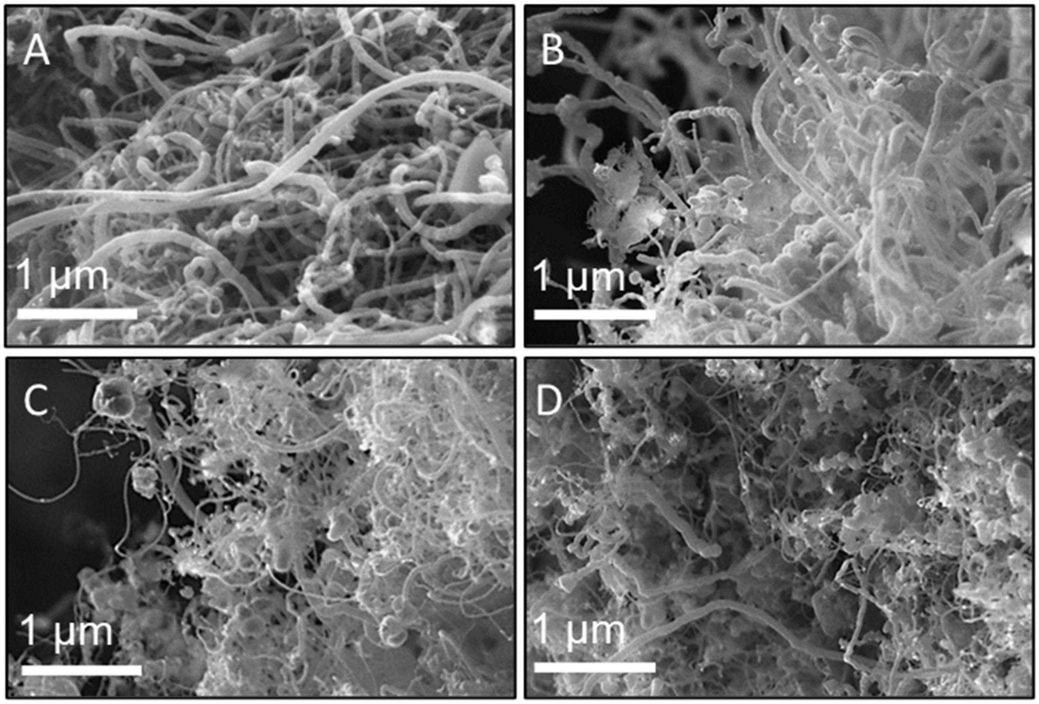


**Figure S4.** SEM images of carbon produced under: (**A**) $CH_4$ feed: (**B**) 95:5 $CH_4$:$CO_2$ vol./vol. feed; (**C**) 99.1:0.9 $CH_4$:$H_2O$ vol./vol. feed; (**D**) 98.4:1.6 $CH_4$:$O_2$ vol./vol. feed.

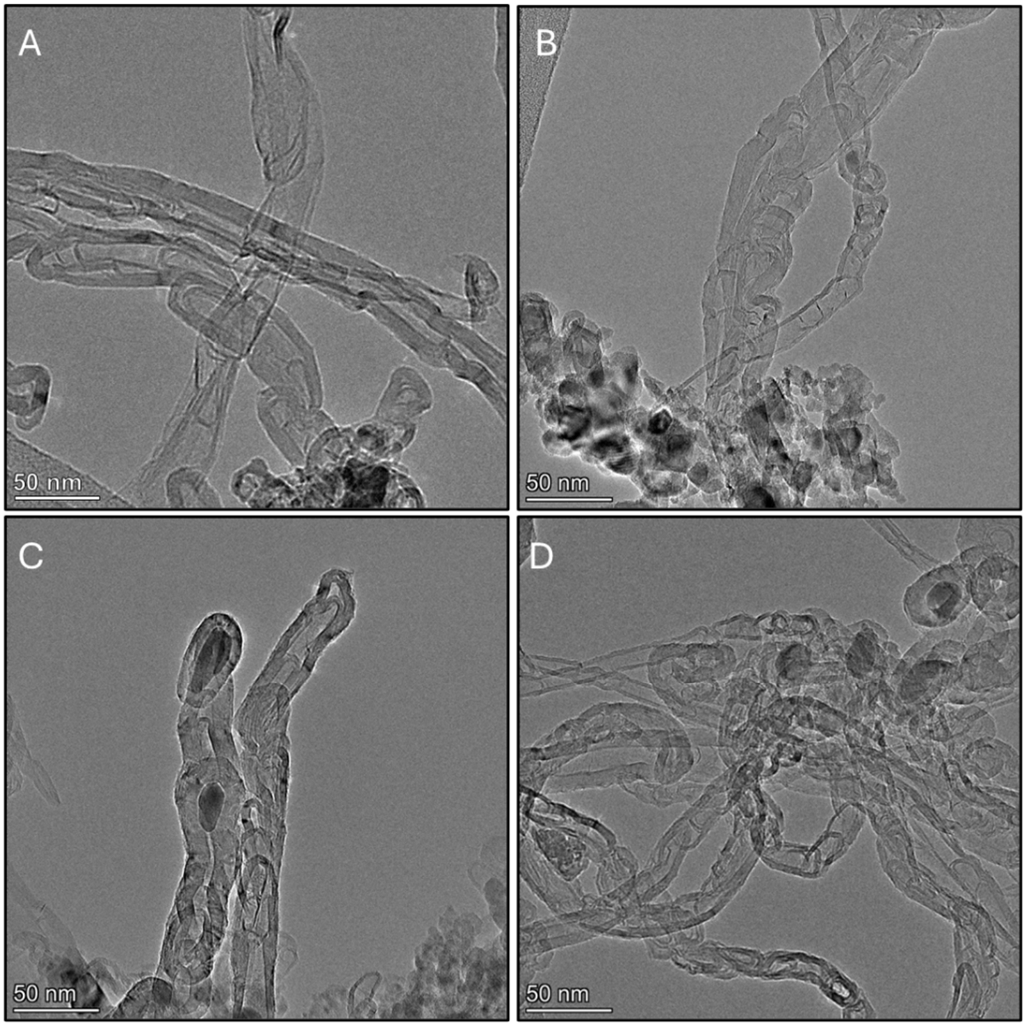


**Figure S5.** TEM images of carbon produced under: (**A**) $CH_4$ feed; (**B**) 95:5 $CH_4$:$CO_2$ vol./vol. feed; (**C**) 99.1:0.9 $CH_4$:$H_2O$ vol./vol. feed; (**D**) 98.4:1.6 $CH_4$:$O_2$ vol./vol. feed.

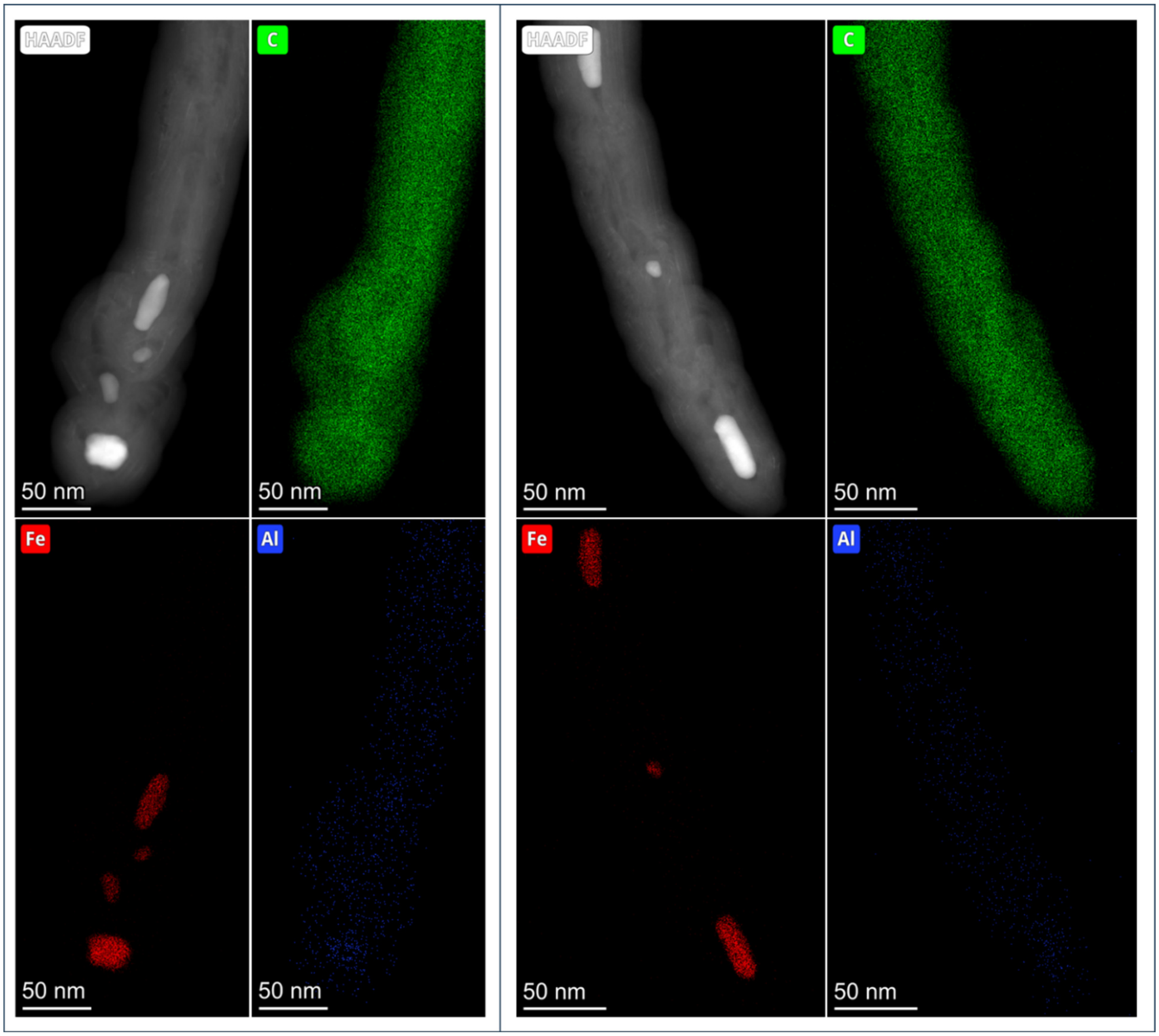


**Figure S6.** TEM-EDS images of CNTs tips showing the spatial distribution of C, Fe and Al. The carbon was produced under 95:5 $CH_4$:$CO_2$ vol./vol. feed.

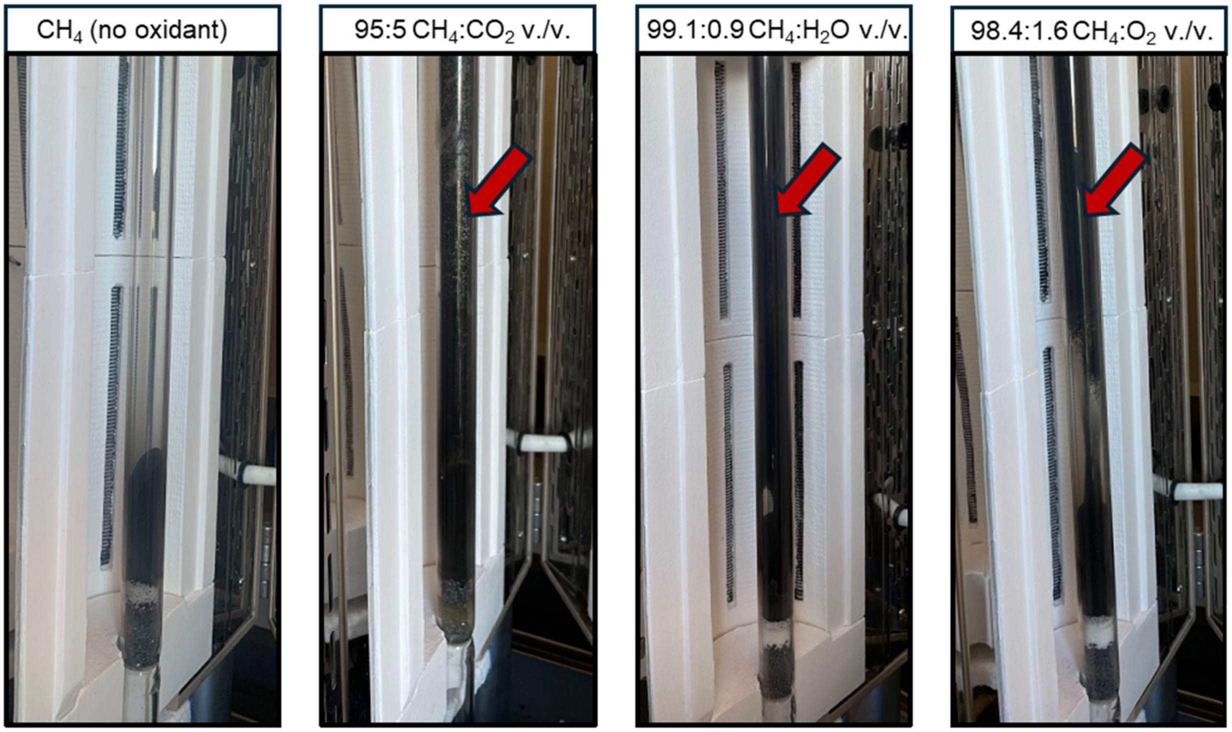


**Figure S7.** Reactor pictures after pyrolysis. A significant amount carbon is accumulated on the reactor walls (indicated by the red arrows) for the cases with an oxidant in the feed.

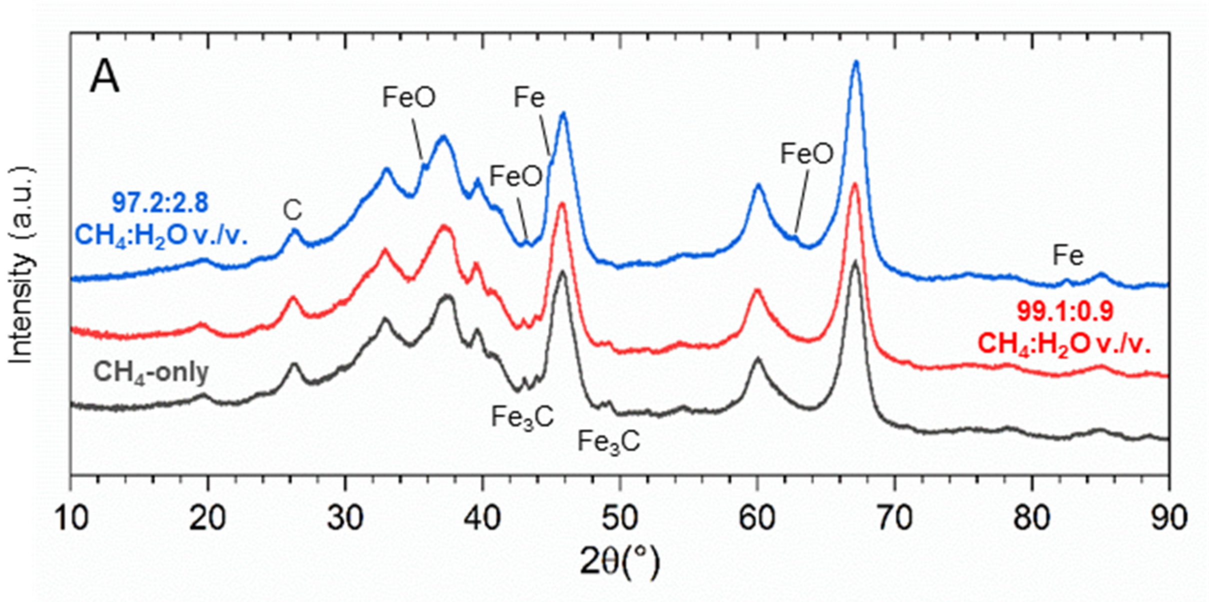


**Figure S8.** XRD spectra of 5 wt% Fe/θ-$Al_2O_3$ catalysts tested under methane-only, 99.1:0.9 $CH_4$:$H_2O$ vol./vol. and 97.2:2.8 $CH_4$:$H_2O$ vol./vol. feed.

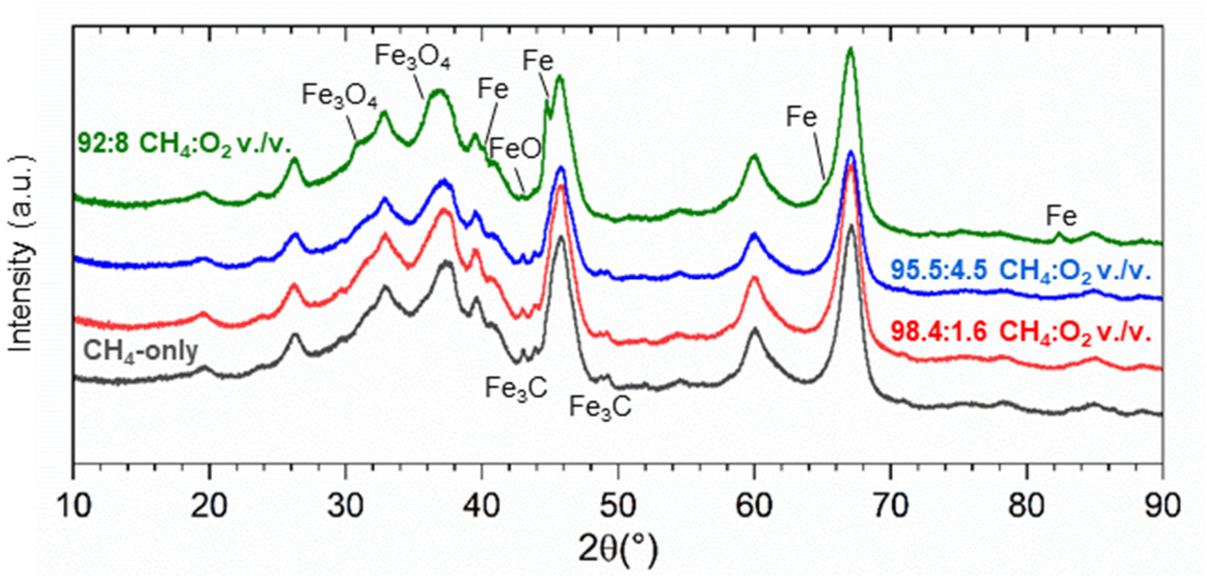


**Figure S9.** XRD spectra of 5 wt% Fe/θ-$Al_2O_3$ catalysts tested under methane-only, 98.4:1.6 $CH_4$:$O_2$ vol./vol., 95.5:4.5 $CH_4$:$O_2$ and 92:8 $CH_4$:$O_2$ vol./vol. feed.

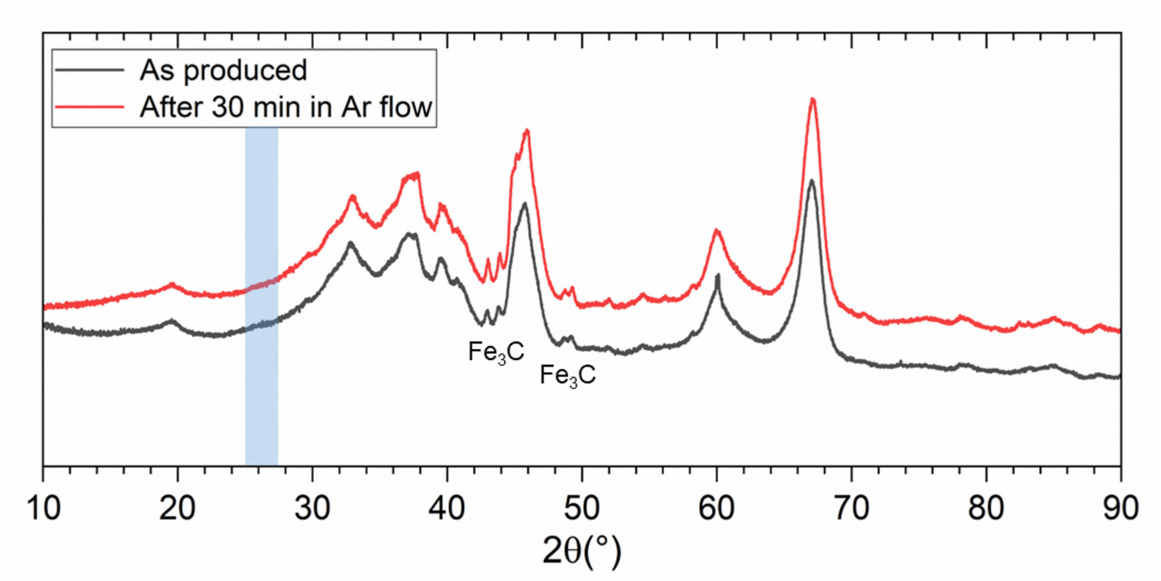


**Figure S10.** XRD spectra of 5 wt% Fe/θ-$Al_2O_3$ catalyst that underwent carburization treatment at 500°C (gray line) and after additional 30 minutes at 750°C under pure Ar (red line). The area highlighted in blue indicates where the peak associated to carbon should be.

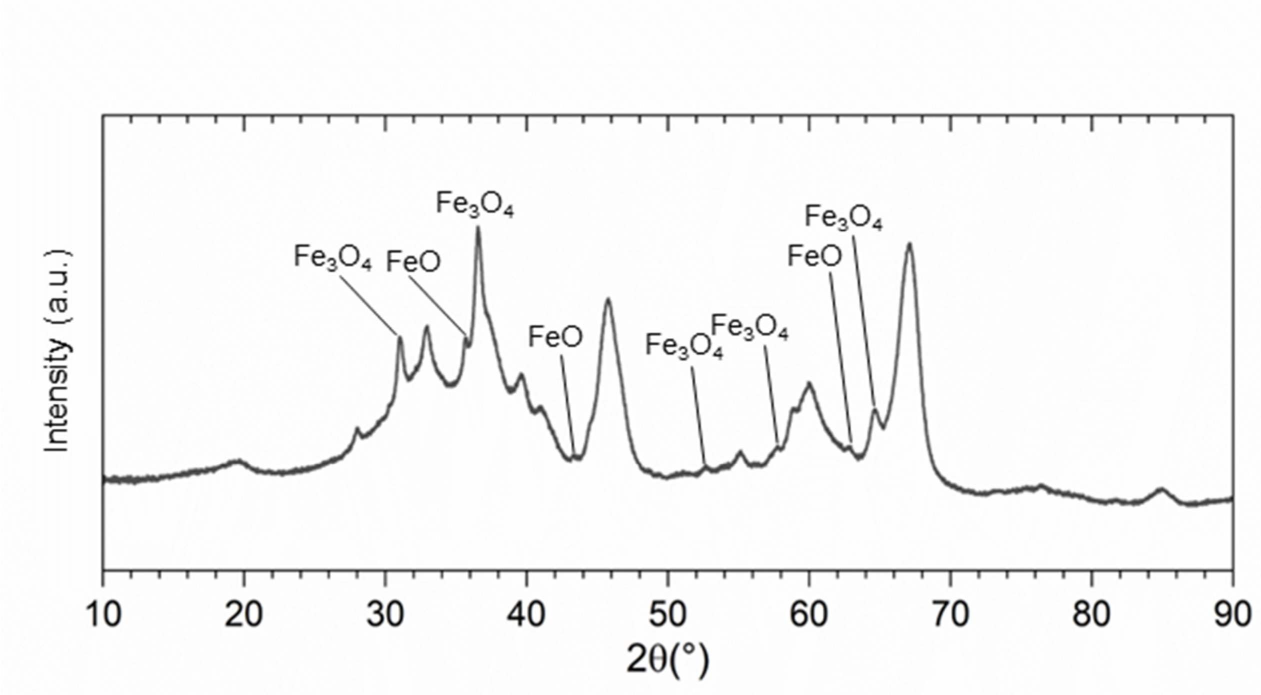


**Figure S11.** XRD spectra of the carburized 5 wt% Fe/θ-$Al_2O_3$ catalyst after 30 minutes at 750°C in 95:5 Ar:$CO_2$ vol./vol.

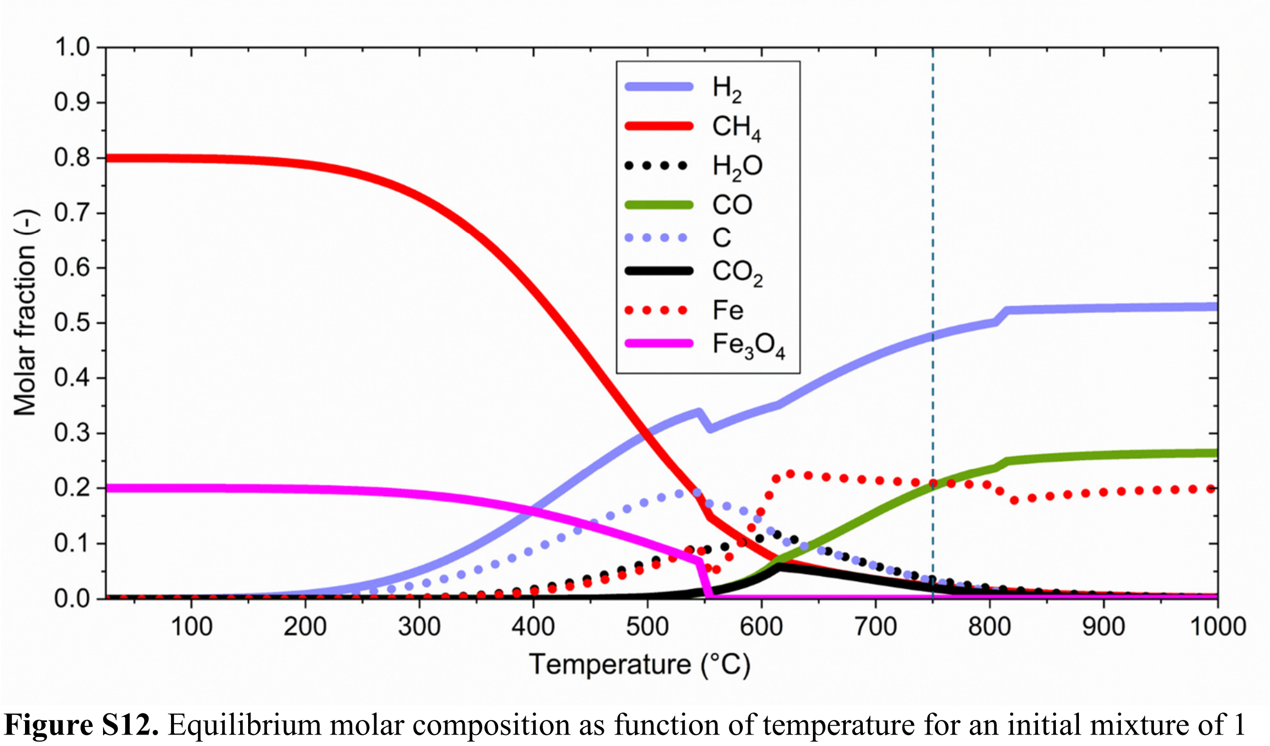


**Figure S12.** Equilibrium molar composition as function of temperature for an initial mixture of 1 mol of $Fe_3O_4$ and 1 mol of $CH_4$ at constant pressure of 1 atm. The vertical dashed line indicates the operating temperature of this work.

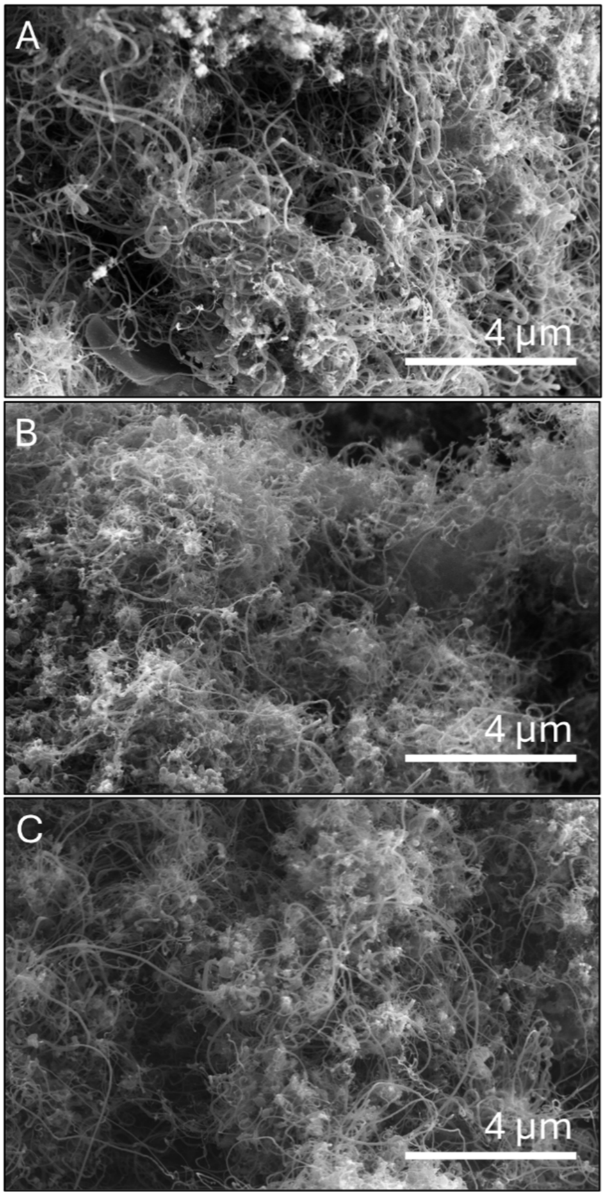


**Figure S13.** SEM images of carbon produced on monolithic catalyst under a: (**A**) $CH_4$ feed; (**B**) 95:5 $CH_4$:$CO_2$ vol./vol. feed; (**C**) 99.1:0.9 $CH_4$:$H_2O$ vol./vol. feed.

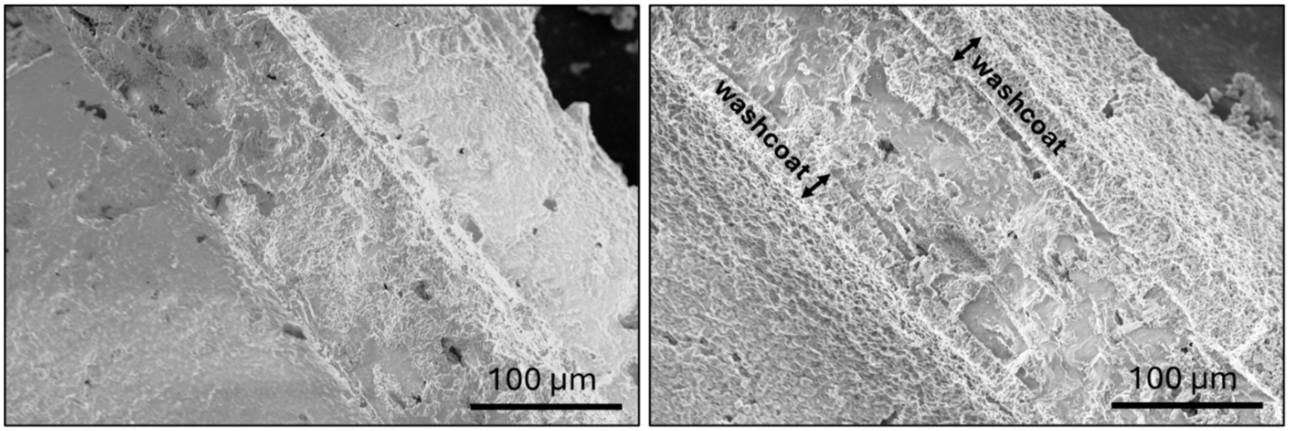


**Figure S14.** SEM images of a cross-section of cordierite monolith before (left) and after (right) the deposition of a porous $Al_2O_3$ layer.

**Technoeconomic assessment of a gas turbine power plant integrated with OMP processes**

Introduction

A gas turbine power plant was selected as a case study to evaluate the potential advantages deriving from its integration with OMP processes. The selected performance metric was the cost of electricity generated by the gas turbine using the fuel produced by OMP with $CO_2$ or $H_2O$, which consists of a blend of hydrogen, carbon monoxide and unreacted methane. For comparison, a similar analysis was performed on the electricity generation costs associated with combusting fuel generated from conventional methane pyrolysis and from direct methane combustion. The modelled performance for the pyrolysis reactor was derived from the rounded experimental data obtained using the monolithic reactor with 95:5 $CH_4$:$CO_2$ vol./vol., 99.1:0.9 $CH_4$:$H_2O$ vol./vol. and $CH_4$-only feeds at 750 °C and 1 bar. The net electrical output of the modelled plant (**Figure S15**) was assumed to be 1 TWh/year in all cases. The process lifetime was modeled as 25 years, with a discount rate of 10%. The TEA covered capital and operating costs for material flows as well as operation and maintenance costs, mainly using literature costing equations(1,2).

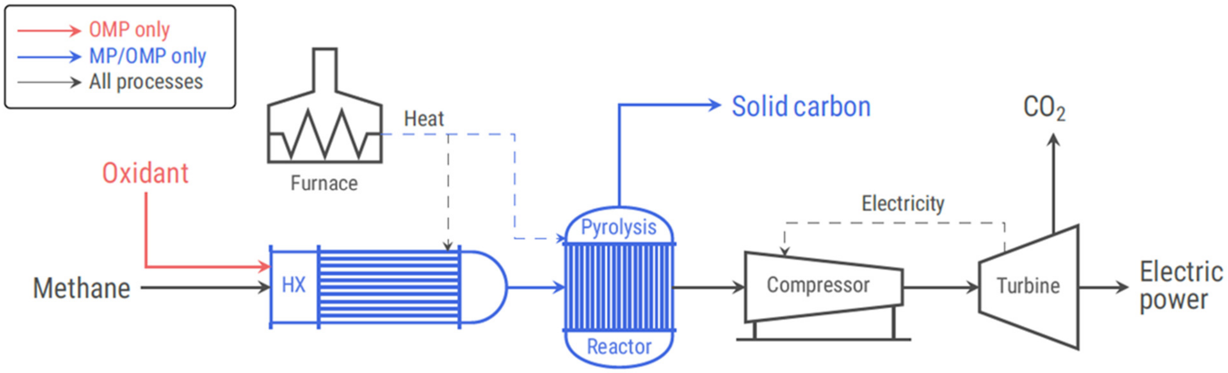

**Figure S15.** Process flowsheet diagram for the four modeled processes that convert methane to electricity and carbon through a series of chemical/physical steps - heating, reaction, cooling, compression, and power generation.

Results and discussion

The levelized cost of electricity generated via the combustion of the gaseous products of $CO_2$ and $H_2O$-assisted methane pyrolysis was estimated to be 32.2 and 31.3 USD/MWh, respectively (**Figure S16**). These values are significantly lower than the cost of electricity obtained via combustion of fuel from conventional methane pyrolysis and pure methane, which are 61.6 and 88.5 USD/MWh, respectively. The reduced cost for the cases with OMP is due to the co-production of larger amounts of solid carbon, which are sold at a conservative cost estimate of 0.6 USD/kg(3).

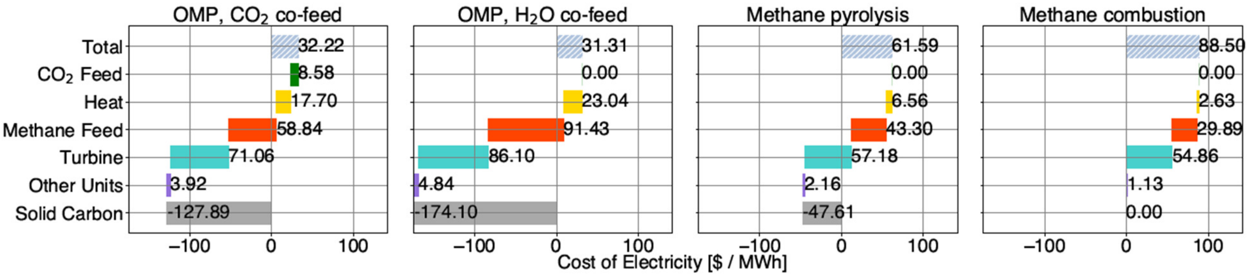


**Figure S16.** A breakdown of the units that contribute to the levelized cost of electricity for each of the four modeled processes, namely power generation via (i) conventional methane combustion, and via combustion of fuel from (ii) conventional methane pyrolysis, and (iii) $CO_2$ and (iv) $H_2O$-assisted methane pyrolysis. The cost of electricity is significantly reduced in the OMP-integrated scenarios since their abundant production of solid carbon revenue serves as a negative cost.

A sensitivity analysis of the impact of carbon value on electricity costs is reported in **Figure S17**. The process economics are significantly dependent on the revenue generated from selling the co-produced solid carbon. Beyond the carbon value of 0.7 USD/$kg_C$, the cost of electricity becomes negative for the OMP-integrated scenarios.

The economic advantages of power generation using OMP fuel depend on the market value of solid carbon. If the solid carbon produced holds a favorable price, the cost savings from OMP fuel can be redirected towards carbon capture, storage, and utilization of direct emissions. This financial flexibility could significantly aid in reaching net-zero emissions in power generation.

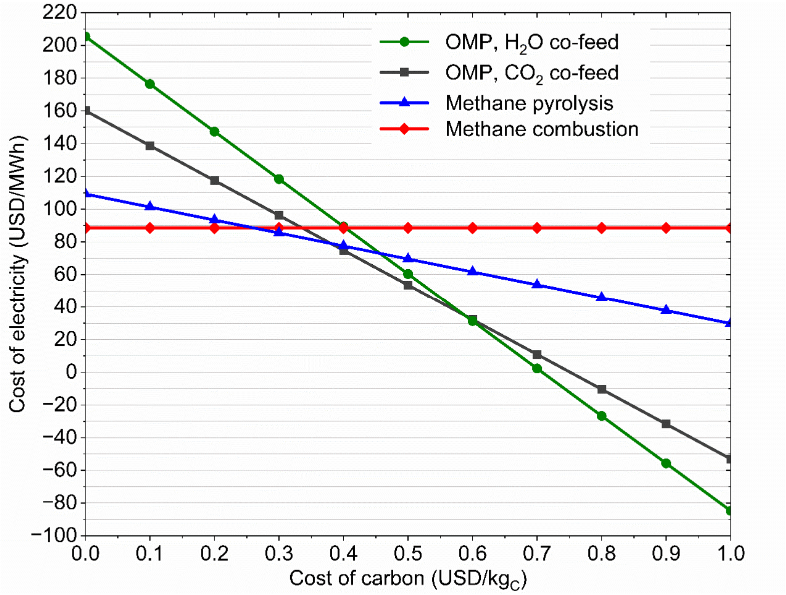


**Figure S17.** Cost of electricity dependence on carbon value.

Methodology

*Pyrolysis reactor mass flows*

The mass flows at the reactor outlets were modeled based on experimental results of the monolithic reactor presented in **Figure 4**. The cases with oxidant co-feed were rounded to a methane conversion rate of 70%, while the case methane-only feed to a 40% methane conversion rate. $CO_2$ and $H_2O$ conversion rates were assumed to be 100%. The oxygen introduced in the pyrolysis reactor in the form of $CO_2$ and $H_2O$ was assumed to convert exclusively into CO. The direct methane combustion case did not include the pyrolysis stage, thus the "reactor outlet" was simply assumed as pure methane. The data utilized is tabulated in **Table S1** for each of the cases.

**Table S1**. Reactor outlet mass per 1 kg of feed input into the reactor for the different scenarios.

| **Outlet Mass [kg]** | ***95:5 $CH_4$:$CO_2$ v./v.*** | ***99.2:0.8 $CH_4$:$H_2O$ v./v.*** | ***Methane pyrolysis*** | ***Methane combustion*** |
|---|---|---|---|---|
| $CH_4$ | 0.26 | 0.30 | 0.6 | 1 |
| $H_2$ | 0.15 | 0.17 | 0.1 | 0 |
| CO | 0.16 | 0.01 | 0 | 0 |
| $CO_2$ | 0 | 0 | 0 | 0 |
| $H_2O$ | 0 | 0 | 0 | 0 |
| $C_{(s)}$ | 0.42 | 0.5 | 0.3 | 0 |

*Heating and electricity demand*

The energy demands - both electricity and heat - were determined for individual units such as compressors, heat exchangers, and reactors. The energy requirement across energy-requiring units

was determined as the difference in enthalpy between inlet and outlet, which was calculated using the thermodynamic software Cantera(4). The heat demand was met by a natural gas-powered industrial furnace, while the electric demand (compressor unit electricity requirement) is satisfied by oversizing the process to cover the electricity demand and still output 1 kWh of net energy generation.

*Overall process metrics*

These process metrics include overall process energy demands and feedstock demands. The direct combustion $CO_2$ emissions (which do not include emissions from natural gas-powered heating) are roughly similar in all cases, despite more sequestered solid carbon in the OMP-integrated cases, since these require more gross energy demand and thus more methane feedstock. The gross energy output consists of the total energy needed to produce a net 1 kWh of output and covers the compressor's energy requirement (i.e. the difference of the gross energy output and 1 kWh is the compressor's energy requirement).

**Table S2**. The process metrics for the different considered scenarios are expressed in terms of a net generation of 1 kWh output, with the exception of the high heating value (HHV).

| **Process Metrics** | **95:5 $CH_4$:$CO_2$ v./v.** | **99.2:0.8 $CH_4$:$H_2O$ v./v.** | **Methane pyrolysis** | **Methane combustion** |
|---|---|---|---|---|
| Reaction outlet HHV [MJ / kg] | 65.71 | 85.17 | 67.82 | 55.50 |
| Direct combustion emissions [$kg_{CO2}$ / net kWh] | 0.49 | 0.47 | 0.44 | 0.50 |
| Sequestered carbon [kgC / net kWh] | 0.21 | 0.29 | 0.08 | 0 |
| Heating demand [MJ / net kWh] | 3.10 | 4.03 | 1.15 | 0.46 |
| $CO_2$ demand [kg / net kWh] | 0.14 | 0 | 0 | 0 |
| $H_2O$ demand [kg / net kWh] | 0 | 0.01 | 0 | 0 |
| Methane demand [kg / net kWh] | 0.36 | 0.56 | 0.26 | 0.18 |
| Gross energy output [kWh / net kWh] | 2.11 | 2.59 | 1.40 | 1.13 |

*Modeling of process units*

The units were sized based on the required balances to produce a net generation of 1 kWh of energy. The capital investment of the units is annualized using a capital recovery factor that is based on a unit economic lifetime of 25 years and an interest rate of 10%. The cost of natural gas is used as a proxy for the cost of methane. Thus, the methane source cost is modeled as the US 2023 annual industrial price average of 4.59 $/10^3 ft^3$, which would amount to 0.25 $/kg for a density of 0.64 kg/m$^3$ at standard temperature and pressure(4–6). The utilized $CO_2$ is modeled to be purchased from industrial point sources, which could cost up to 60 $/t$_{CO2}$ in some industries(7). Although it would be much more environmentally beneficial to utilize atmospheric $CO_2$, the cost of direct air capture remains significantly high(8). Water purchases cost 0.2 $ / m$^3$, which is around $2 \cdot 10^{-4}$ $ / kg$_{H2O}$ (9). The industrial furnace is modeled via capital and operating costs.

The capital cost of the natural gas furnace is expressed as(10):

$$C_{furnace}^{capex} = 250 \cdot \dot{Q}$$

where $C_{furnace}^{capex}$ is the furnace capital cost as a function of the associated heat load $\dot{Q}$.

Furthermore, the variable natural gas cost is expressed as(10):

$$C_{furnace}^{opex} = 7.2 \cdot \dot{Q} + c_{NG} \cdot Q + c_{DPE} \cdot m_{DPE}$$

where $C_{furnace}^{opex}$is the furnace operating costs, which are modeled as a function of the annual heat energy generated, $Q$, the cost of natural gas, $c_{NG}$, which is 4.59 $/MMBtu(5), and $c_{DPE}$ the cost of the diphenyl ether (DPE), priced at 84 CHF/kg(11). DPE is used as the heating fluid due to its high temperature range as a gas before reaching its critical state(12). In addition, a fixed operating and maintenance cost of 4% of the CAPEX is modeled.

The compressor work is determined by(13):

$$\dot{W}_{compressor} = \frac{\dot{m} \cdot w_{compressor}^{is}}{\eta_{compressor}^{is} \cdot \eta_m \cdot \eta_g}$$

where $\dot{m}$ is the mass flow across the compressor, $w_{compressor}^{is}$ is the isentropic specific work of the unit (generated from Cantera), $\eta_{compressor}^{is}$ is the isentropic efficiency (80%), $\eta_m$ is the mechanical efficiency (98%), and $\eta_g$ is the electric generator efficiency (96%)(13). The isentropic specific work is determined using the enthalpy difference across an isentropic increase in the pressure(4). The unit-specific electricity demand to isentropically increase the reaction outlet pressure from 1 bar to 20 bar is shown for each of the 4 processes in **Table S3**.

**Table S3**. The unit-specific electricity demand ($w_{compressor}^{is}$) for the compressor associated with each process.

| | **95:5 $CH_4$:$CO_2$ v./v.** | **99.2:0.8 $CH_4$:$H_2O$ v./v.** | **Methane pyrolysis** | **Methane combustion** |
|---|---|---|---|---|
| Electricity demand [kWh / kg] | 1.666 | 2.125 | 1.1248 | 0.5186 |

The compressor cost is based on its power rating, $\dot{W}_{compressor}$(1):

$$C_{compressor} = 580000 + 20000 \cdot \dot{W}_{compressor}^{0.6}$$

where $C_{compressor}$ is the compressor capital cost of the compressor.

The heat exchanger units encompass coolers and heaters. A heat exchanger (HX) is modeled as a tube and shell heat exchanger, in which the heating/cooling fluid is diphenyl ether/ammonia.

Heat exchangers in the methane pyrolysis processes are used for increasing the feed to the reaction temperature of 750 °C. No heat integration is assumed in this model.

The heat load required is a function of many factors such as the heat exchanger design, the involved fluids and their flowrates. The analysis centers on the heat needed to reach desired temperatures, which is calculated using Cantera(4), as well as the heat energy required to reach the final temperature for 1 kg of the inlet. Based on the amount of mass to be heated in one year, which is determined from the process functional unit and the scale factors, the energy quantity $Q$ is transformed into the heat rate, $\dot{Q}$. The unit-specific heat demand to raise the feeds' temperature from 25 °C to 750 °C at 1 bar is shown for each of the 4 processes in **Table S4**.

**Table S4**. The unit-specific heat demand for the heat exchanger associated with each process.

| | **95:5 $CH_4$:$CO_2$ v./v.** | **99.2:0.8 $CH_4$:$H_2O$ v./v.** | **Methane pyrolysis** | **Methane combustion** |
|---|---|---|---|---|
| Heat demand [MJ / kg] | 2.297 | 2.507 | 2.517 | 2.517 |

The heat exchanger is sized based on Kern's methodology for a shell and tube heat exchanger(14). The cost of this heat exchanger depends on its area(1):

$$C_{HX} = 28000 + 54 \cdot A_{HX}^{1.2}$$

where $C_{HX}$ is to the heat exchanger cost and $A_{HX}$ is the heat exchange surface area(2).

The turbine herein consists of a combustor of the pyrolysis reactor outlet fuel and a gas turbine. The combustor generates direct $CO_2$ emissions and requires a stoichiometric oxygen feedstock input. The oxygen price is 40 $/$t_{O2}$(15). The combustion emissions are estimated based on the oxidation of all C-carrying molecules into C. Thus, the direct emissions are dependent on the composition of the reaction outlet streams.

The gas turbine operates at a high heating value efficiency of 40% and requires a feed of 20 bars(16). The turbine cost is derived based on gas turbine capital costs, which according to the NREL annual technology baseline, consists of 1329 $/kW installed capacity(17). Its annual fixed operation and maintenance costs are assumed to be 4% of the capital cost(2).
The modeling of the reactor size is based on the reaction data, which is used to yield the reactor empty volume, $\forall_{RX}$, which is determined by:

$$\forall_{RX} = \frac{\dot{m}_{RX}}{\beta \cdot \rho_{cat} \cdot WHSV}$$

where $\dot{m}_{RX}$ is the mass flowrate across the reactor, $\beta$ is the porosity fraction (assumed to be 0.4(2)) and $\rho_{cat}$ is the catalyst density, and $WHSV$ is the weight hourly space velocity, which quantifies the mass flowrate that can be catalyzed by a unit mass of catalyst. The conditions associated with each reaction are summarized in **Table S5**.

**Table S5**. The various reactions considered for the modeled processes. The table highlights the reaction yields such as selectivity ($\varsigma$), conversion ($\chi$), based on the reaction conditions such as temperature (T), pressure (p), weight hourly space velocity (WHSV), and the utilized catalyst. No selectivity values are reported for the methane pyrolysis co-feed reactions since the utilized data was experimentally measured data.

| Reaction | $\varsigma$ [%] | $\chi$ [%] | WHSV [$h^{-1}$] | T [°C] | P [bar] | Catalyst |
|---|---|---|---|---|---|---|
| OMP | - | 70 | 14.5 | 750 | 1 | $Fe/Al_2O_3$ |
| MP | - | 40 | 14.5 | 750 | 1 | $Fe/Al_2O_3$ |

The reactor capital cost is determined based on its volume(1):

$$C_{RX}^{capex} = 61500 + 32500 \cdot \forall_{RX}^{0.8}$$

Where $C_{RX}^{capex}$ is the reactor capital cost and $\forall_{RX}$ is the reactor volume. The costing of the reactor also includes its catalysts, which are modeled as the reactor operating cost:

$$C_{RX}^{opex} = c_{cat} \cdot \frac{\dot{m}_{RX}^{rec}}{WHSV}$$

Where $C_{RX}^{opex}$ is the reactor operating cost and $c_{cat}$ is the specific catalyst cost. The catalysts are replenished every year, and their costs are included as a yearly operating cost.

The catalyst costs are sourced from Thermo Fisher Scientific(18) and are determined as weighted (mass fraction-based) average of the individual catalyst metal costs. In this case, the catalyst is modeled as a 5/95 percentage of mass split between iron and alumina, whose costs per kg are respectively 2 and 156 $/kg.

Pyrolysis reactors also require heat since the reactions are endothermic, and thus, the unit-specific heat demand to maintain the reaction at 750 °C at 1 bar is shown for each of the 4 processes in **Table S6**.

**Table S6**. The unit-specific heat demand for the pyrolysis reactors associated with each process. Methane combustion requires no heat demand since it does not include a pyrolysis reaction in this process(19).

| | **95:5 $CH_4$:$CO_2$ v./v.** | **99.2:0.8 $CH_4$:$H_2O$ v./v.** | **Methane pyrolysis** | **Methane combustion** |
|---|---|---|---|---|
| Heat demand [MJ / kg] | 3.8614 | 4.6347 | 1.82 | 0 |

## References


1. Towler G, Sinnott R. Capital Cost Estimating. *Chemical Engineering Design*. 2013; 307–54.
2. Saad DM, Terlouw T, Sacchi R, Bauer C. Life Cycle Economic and Environmental Assessment of Producing Synthetic Jet Fuel Using $CO_2$/Biomass Feedstocks. *Environ Sci Technol*. 2024; 58(21):9158–74.
3. Zhang J, Liang C, Dunn JB. Graphite Flows in the U.S.: Insights into a Key Ingredient of Energy Transition. *Environ Sci Technol*. 2023; 57(8):3402–14.
4. Cantera: An Object-oriented Software Toolkit for Chemical Kinetics, Thermodynamics, and Transport Processes. Available from: https://zenodo.org/records/8137090
5. Henry Hub Natural Gas Spot Price (Dollars per Million Btu). Available from: https://www.eia.gov/dnav/ng/hist/rngwhhdM.htm
6. U.S. Natural Gas Prices. Available from: https://www.eia.gov/dnav/ng/ng_pri_sum_dcu_nus_a.htm
7. Hughes S, Zoelle A, Woods M, Henry S, Homsy S, Pidaparti S, et al. Cost of Capturing $CO_2$ from Industrial Sources. 2022; Available from: https://www.osti.gov/servlets/purl/1887586/
8. Sievert K, Schmidt TS, Steffen B. Considering technology characteristics to project future costs of direct air capture. *Joule*. 2024; 8(4):979–99.
9. Seider WD., Lewin DR., Seader JD., Widagdo Soemantri, Gani R., Ng KM. *Product and process design principles: synthesis, analysis, and evaluation*. John Wiley & Sons. (2017).
10. Popovski E, Fleiter T, Santos H, Leal V, Fernandes EO. Technical and economic feasibility of sustainable heating and cooling supply options in southern European municipalities-A case study for Matosinhos, Portugal. *Energy*. 2018; 153:311–23.
11. Diphenylether ≥99%, FG | Sigma-Aldrich. Available from: https://www.sigmaaldrich.com/CH/de/product/aldrich/w366706
12. Chickos JS, Hosseini S, Hesse DG. Determination of vaporization enthalpies of simple organic molecules by correlations of changes in gas chromatographic net retention times. *Thermochim Acta*. 1995; 249:41–62.
13. Minutillo M, Perna A, Forcina A, Di Micco S, Jannelli E. Analyzing the levelized cost of hydrogen in refueling stations with on-site hydrogen production via water electrolysis in the Italian scenario. *Int J Hydrogen Energy*. 2021; 46(26):13667–77.
14. Flynn AM, Akashige T, Theodore L. Shell-and-Tube Heat Exchangers. *Kern's Process Heat Transfer*. John Wiley & Sons. 2019; 289–380.
15. Dorris CC, Lu E, Park SJ, Toro F. High-Purity Oxygen Production Using Mixed Ionic-Electronic Conducting Sorbents. *Senior Design Reports (CBE)*. 2016;
16. Darabkhani HG, Varasteh H, Bazooyar B. An introduction to gas turbine systems. *Carbon Capture Technologies for Gas-Turbine-Based Power Plants*. 2023; 1–18.
17. Data | Electricity | 2024 | ATB | NREL. Available from: https://atb.nrel.gov/electricity/2024/data
18. Catalysts | Thermo Fisher Scientific - US. Available from: https://www.thermofisher.com/us/en/home/chemicals/inorganic-chemistry/catalysts.html

19. Goodwin DG, Speth RL, Moffat HK, Weber BW, Goodwin DG, Speth RL, et al. Cantera: An Object-oriented Software Toolkit for Chemical Kinetics, Thermodynamics, and Transport Processes. *Zenodo*. 2021; Available from: https://ui.adsabs.harvard.edu/abs/2021zndo...4527812G/abstract
